# Lepidopteran biodiversity of Ethiopia: current knowledge and future perspectives

**DOI:** 10.3897/zookeys.882.36634

**Published:** 2019-10-23

**Authors:** Tesfu Fekensa Tujuba, Andrea Sciarretta, Axel Hausmann, Getnet Atnafu Abate

**Affiliations:** 1 Ethiopian Biodiversity Institute, Addis Ababa, Ethiopia Ethiopian Biodiversity Institute Addis Ababa Ethiopia; 2 Department of Agriculture, Environment and Food Sciences, University of Molise, Campobasso, Italy University of Molise Campobasso Italy; 3 SNSB-Bavarian State Collection of Zoology, Munich, Germany Bavarian State Collection of Zoology Munich Germany; 4 Department of Biology, Debre Markos University, Debre Markos, Ethiopia Debre Markos University Debre Markos Ethiopia

**Keywords:** Africa, butterflies, checklist, DNA barcoding, endemic, Ethiopian moths

## Abstract

Lepidoptera is the second largest order of insects. Encompassing moths and butterflies, it is regarded as one of the most important components of biodiversity. Here, an updated comprehensive overview of Lepidoptera recorded in Ethiopia is presented, composed of 2,438 taxa in 48 families, of which 664 are endemic. Records were compiled from various literature sources and website databases. Although still being far from complete, this review provides important baseline data for understanding zoogeographic patterns and thus for undertaking effective conservation action. Further research on Ethiopian Lepidoptera is encouraged.

## Introduction

Ethiopia is among the largest countries in the African continent, located in the horn of Africa, covering a total area of 1,127,127 km^2^ (Gordon and Carillet 2003; EBI 2015; Tesfu et al. 2018). It belongs to the Afro-tropical Region (former Ethiopian Region) and, based on the bioclimatic classification of Burgess et al. (2004), comprises the zones “Sahelian Savanna”, “Somalian Xeric Bushland and Shrubland” and “Ethiopian Montane forest and Alpine Moorland” (Hacker 2019). The country’s topography is very diverse, with 20 mountains peaks above 4,000 meters. The highest mountain, Ras Dashen, peaks 4,620 m above sea-level, the fourth-highest in Africa, whilst the third-lowest point in Africa, the Danakil Depression, reaches down to 125 m below sea level. The dominating topographic element is the vast and fertile central highland that accounts for 37% of the land area of the country with an average elevation from 1,500 to 2,400 m that deserved the country to be known as ‘roof of Africa’. It is the largest block of land above 1,500 m in Africa (Clausnitzer and Dijkstra 2005), dissected by the Great Rift Valley and surrounded by lowlands along the periphery (Gordon and Carillet 2003). The mean annual rainfall ranges from 500 mm to 2,800 mm and the mean annual temperatures range from around 10° to above 30 °C. Because of these diverging abiotic parameters, the country is endowed with an amazingly diversity of plant, animal and microbial organisms (EBI 2015). According to Clausnitzer (2014), the rate of endemism in Ethiopia’s ﬂora and fauna is exceptionally high as a result of vast highlands being isolated by the surrounding dry lowlands. Only the most eurytopic and mobile species (usually those of the lowlands) tend to be found in both Ethiopia and the rest of tropical Africa. In the same manner, Kravchenko et al. (2007) stated that the territory of Ethiopia hosts an extraordinarily diverse landscape including high mountains, lowlands, deserts and tropical rain forests that resulted in a hyperdiverse fauna and flora. Likewise, in consequence of its rich biodiversity, Ethiopia is acknowledged as one of the 20-mega-biodiverse countries in the world (Mittermeier et al. 2011; Tesfu et al. 2018).

Lepidoptera represent the second largest insect order, which consists of approximately 140 different families and 160,000 species that have been described and recognised worldwide, so far (Biodiversity Institute of Ontario 2006; Kristensen et al. 2007; Nieukerken et al. 2011). Lepidoptera comprise nearly 17% of all insect species, and some recent estimates suggest that the real number of Lepidoptera species would set up to 500,000 species (Brandao et al. 2009).

The aims of this paper are to give an updated comprehensive presentation of the actual knowledge of Ethiopian Lepidoptera and to provide some estimates for the expected biodiversity of this major insect order in the country.

## Materials and methods

The present review is based on all pertinent published scientific papers. In addition, records from up to date and relevant online databases were also included, particularly, records from the Natural History Museum of London website (“NHMUK”: Beccaloni et al. 2003), the Barcode of Life Data Systems (“BOLD”: Ratnasingham and Hebert 2007), the African moth website (Goff 2008), LepiMap (Navarro 2007), the African Butterfly Database (Sáfián et al. 2009), the Afromoth website (De Prins and De Prins 2019) and the Afrotropical Butterflies and Skippers digital encyclopaedia (Williams 2018). In all cases, records were included only when sample identifications were made at specific (or subspecific) level, and the provenience from Ethiopia was clearly indicated. Data from entomological collections but not publicly accessible were not considered.

We followed the classification system and nomenclature (valid names and synonymies) used in De Prins and De Prins (2019) with some updates coming from more recent publications. For Rhopalocera, the Afrotropical Butterflies and Skippers digital encyclopaedia (Williams 2018) served as reference. These two outstanding references have also represented the fundamental database and resource for our compilation of the lepidopteran fauna of Ethiopia.

## Lepidoptera exploration in Ethiopia: from early explorers to present

Many entomologists have contributed to our current knowledge of the Ethiopian Lepidoptera fauna. The following selection provides the most significant contributions made by past pioneers and current explorers.

Johann Christoph Friedrich Klug in 1829 was the first to mention Abyssinia, the former name of Ethiopia, in the description of a new Lepidoptera species, the butterfly *Pontia eupompe* (Klug, 1829) now Colotis danae ssp. eupompe (Nazari et al. 2011), indicating as locus typicus “in Arabia deserta, in Sinai monte, in Dongala et Habessinia”.

From the mid-nineteenth century, additional descriptions came from few authors such as Félix Edouard Guérin-Méneville (1849), Louis Reiche (1850), and Hippolyte Lucas (1852). However, the most significant advance in the nineteenth century was made by the French entomologist Achille Guenée, who published various contributions between 1852 and 1858. He described 31 new species belonging to the Noctuoidea and Geometroidea, based on material collected mainly by Georg Wilhelm Schimper in 1850. In all cases, the locus typicus was indicated as “Abyssinia” (Guénée 1852).

Other important contributions to the study of Ethiopian Lepidoptera were made subsquently, many of which have reported the description of new species from specimens collected in the country. For instance, George Hampson described 23 species from different families in the period between 1896 and 1930 (Hampson 1896, 1898, 1899, 1905, 1909, 1910, 1913, 1916, 1918, 1919, 1926, 1930). Edward Meyrick firstly reported Microlepidoptera from the country, with 40 new species, from the material collected during the expeditions carried out by Hugh Scott and Omer-Coper in 1926–1927 (Meyrick 1932). The most important contribution to the study of butteflies was made by Lionel Walter Rothschild and Karl Jordan, during the first decades of the twentieth century, with 34 new taxa (Rothschild 1902, 1926; Rothschild and Jordan 1900, 1903, 1905). Debauche (1937) reported 42 geometrid species from Ethiopia with eight new descriptions. Likewise, Emilio Berio published many papers dedicated to the Erebidae and Noctuidae of East Africa, describing from Ethiopia 12 and 37 species, respectively (Berio 1939a, 1940a, 1943, 1944, 1945, 1947, 1954, 1962, 1975, 1977), some of them from the localities of Adu-Abuna and Metema, at that time part of Eritrea, but now in Tigray, Northern Ethiopia (Berio 1937, 1939b, 1939c, 1940b, 1973, 1976). Pierre-Claude Rougeot has explored the country several times in 1970s and described 55 new species belonging to various families (Rougeot 1974, 1975, 1977, 1984, Plantrou and Rougeot 1979; Laporte and Rougeot 1981; Rougeot and Laporte 1983). The two French entomologists Bernard Laporte and Claude Herbulot in their publications described from Ethiopia 137 new noctuid (specifically, eight species of Erebidae, two species of Nolidae, and 127 Noctuidae) and 22 new geometrid taxa, respectively (Herbulot 1983, 1993, 2002; Laporte 1974, 1975, 1976, 1978; Rougeot 1977, 1984; Laporte and Rougeot 1981; Rougeot and Laporte 1983; Rougeot et al. 1991).

With the new millennium, the country has awakened a renewed interest from entomologists, which led to the description of 255 new taxa in 18 years. In particular, major contributions to Ethiopian Lepidoptera were made by Hermann H. Hacker, with various colleagues, for Erebidae, Nolidae and Noctuidae (178 new taxa); David Agassiz for Yponomeutidae (five new taxa); Jósef Razowski and Pasquale Trematerra for Tortricidae (34 new taxa described); Axel Hausmann, Andrea Sciarretta and Francesco Parisi for Geometridae (27 new taxa); Ulf Eitschberger and Tomas Melichar for Sphingidae, with eleven new taxa (Hacker and Fibiger 2007; Hacker and Zilli 2007; Haxaire and Melichar 2008; Hausmann et al. 2014, 2016; Hacker et al. 2008, 2012; Razowski and Trematerra 2010, 2012; Hacker and Mey 2010; Hacker 2011, 2013, 2014, 2016, 2019; Eitschberger and Ströhle 2011; Melichar and Řezáč 2015; [Bibr B27][Bibr B77]; Razowski et al. 2018; Agassiz 2019).

Many of these and other minor contributions resulted from dedicated expeditions, such as the “Joint Ethiopian-Russian Biological Expedition” lead by Vasiliy Kravchenko from Tel Aviv University, Israel; the “Ethiopian Insects Project”, between the Ethiopian Wildlife Conservation Authority (EWCA), the Bavarian State Collection of Zoology (ZSM) and the Museum Thomas Witt (MWM) in Munich, Germany; the projects carried out by the Italian entomologists of the University of Molise with EWCA and Ethiopian Biodiversity Institute (Kravchenko et al. 2007; Sciarretta et al. 2014; Hausmann et al. 2016).

## Current state of knowledge on Ethiopian Lepidoptera

Based on the results of our current review, 2,438 Lepidoptera taxa (species or subspecies) are known to occur in Ethiopia hitherto, belonging to 48 families (Table 1; full list at: https://doi.org/10.5281/zenodo.3234617). This number includes 170 taxa which are not reported by the scientific literature but have been extracted from the above-mentioned websites.

**Table 1.** Ethiopian Lepidoptera families and number of taxa (species and subspecies) reported.

No.	Family	Total number of taxa	Common name
1	Bedelliidae	1	Narrow-winged moths
2	Blastobasidae	2	Scavenger moths
3	Bombycidae	2	Silkworm moths
4	Brahmaeidae	2	Brahmin moths
5	Carposinidae	1	Fruitworm moths
6	Choreutidae	2	Metalmark moths
7	Cosmopterigidae	4	Cosmet moths
8	Cossidae	17	Carpenterworm moths
9	Crambidae	109	Grass moths
10	Depressariidae	2	Flat-bodied moths
11	Drepanidae	1	Hook-tips
12	Elachistidae	1	Grass miner moths
13	Epermeniidae	1	Fringe-tufted moths
14	Erebidae	523	Tiger moths
15	Eupterotidae	8	Snout moths
16	Euteliidae	10	Euteliid moths
17	Gelechiidae	10	Twirler moths
18	Geometridae	306	Geometer moths
19	Glyphipterigidae	1	Sedge moths
20	Gracillariidae	13	Leafminer moths
21	Hesperiidae	36	Skipper butterflies
22	Hyblaeidae	1	Teak moths
23	Lasiocampidae	38	Lappet moths
24	Limacodidae	15	Slug caterpillar moths
25	Lycaenidae	116	Gossamer-winged butterflies
26	Lyonetiidae	2	Lyonet moths
27	Metarbelidae	4	Wood-borer moths
28	Noctuidae	471	Owlet moths
29	Nolidae	85	Tuft moths
30	Notodontidae	28	Prominent moths
31	Nymphalidae	178	Brush-footed butterflies
32	Oecophoridae	1	Concealer boths
33	Papilionidae	17	Swallowtail butterflies
34	Pieridae	79	Yellows, Whites, & Sulphurs
35	Plutellidae	5	Diamondback moths
36	Psychidae	6	Bagworm moths
37	Pterophoridae	39	Plume moths
38	Pyralidae	31	Snout moths
38	Saturniidae	53	Emperor moths
40	Scythrididae	7	Flower moths
41	Sesiidae	6	Clearwing moths
42	Sphingidae	81	Hawk moths
43	Thyrididae	9	Picture-winged leaf moths
44	Tineidae	38	Fungus moths
45	Tortricidae	60	Leafroller moths
46	Uraniidae	3	Swallowtail moths
47	Yponomeutidae	6	Ermine moths
48	Zygaenidae	8	Burnet moths
**Total**		**2438**	

In particular, 929 species or subspecies were described from type specimens collected in Ethiopia, 131 of them, mostly butterflies, at subspecific level (Table 2). It is interesting to note that endemic taxa number 664, approximately 27% of the total Lepidoptera. This high number can be explained by the particular physical and biogeographical history of the country and a broad range of different ecosystems with great diversity of habitats.

**Figures 1–6.**
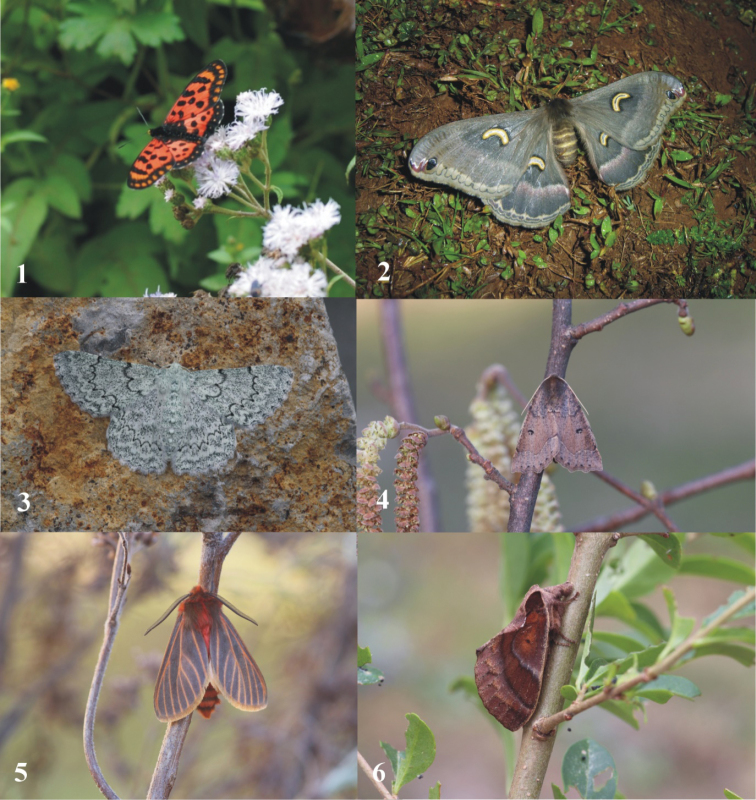
**1***Acraea oscari* Rothschild, 1902 **2***Epiphora fournierae* (Le Moult, 1945) **3***Pingasa pallidata* (De Joannis, 1913) **4***Odontopera protecta* Herbulot, 1983 **5***Metarctia flavivena* Hampson, 1901 **6***Stoermeriana laportei* (Rougeot, 1977). Photos credit: Alenuccio Palladino (**1**); Francesco Parisi (**2**); Dirk Stadie (**3–6**).

**Table 2.** List of Lepidoptera taxa originally described from Ethiopia (only valid names are listed). An asterisk (*) denotes that the town is in Sudan, but the river originates in Ethiopia. The type locality is recorded with the corrected spelling or current locality name in square brackets. The endemic taxa from Ethiopia are indicated with E in the last column. Synonymies are not reported. De Prins and De Prins (2019) and Williams (2018) have been used as a basic reference for the preparation of the list.

	Family	Taxon	Author	Type Locality	
1	Blastobasidae	*Blastobasis eridryas*	Meyrick, 1932	Mt Chillálo	E
2	Brahmaeidae	*Dactyloceras richinii*	Berio, 1940	Adi Abuna [in Tigray, Ethiopia]	
3	Carposinidae	*Carposina candace*	Meyrick, 1932	Jem-Jem Forest	E
4	Choreutidae	*Choreutis argyrastra*	Meyrick, 1932	Mt Zukwala/Cuqala	E
5		*Telosphrantis aethiopica*	Meyrick, 1932	Mt Chillálo	E
6	Cosmopterigidae	*Ascalenia secretifera*	Meyrick, 1932	Mt Chillálo	E
7		*Cosmopterix derrai*	Koster, 2016	14 km S of Debre Tabor, Alemsaga Forest	E
8		*Cosmopterix epismaragda*	Meyrick, 1932	Jem-Jem Forest	E
9	Cossidae	*Aethalopteryx obscurascens*	(Gaede, 1930)	Centr. Abyss., Maraquo	
10		*Afroarabiella strohlei*	Yakovlev & Witt, 2016	Turmi, Mango Lodge	E
11		*Azygophleps brehmi*	Yakovlev & Witt, 2016	Bale Mountains, Karcha near Rira	E
12		*Camellocossus abyssinica*	(Hampson, 1910)	Abyssinia [Ethiopia]	
13		*Camellocossus lalibela*	Yakovlev & Witt, 2017	Arba Minch	E
14		*Camellocossus strohlei*	Yakovlev & Witt, 2017	Arba Minch	E
15		*Macrocossus sidamo*	Rougeot, 1977	near Kébré-Mengist [Kebre Mengist]	E
16		*Oreocossus ungemachi*	Rougeot, 1977	Ioubdo, Birbir	E
17		*Strigocossus kushit*	Yakovlev, 2011	Ethiopia SE, Bale, 11 km SW Goba, Bale Mts	E
18	Crambidae	*Adelpherupa aethiopicalis*	Maes, 2002	SW Abyssinia [Ethiopia], Djimma [Jimma]	E
19		*Agathodes dufayi*	Rougeot, 1977	Koffolé [Kofale]	E
20		*Alphacrambus cristatus*	Bassi, 1995	Maraqo	E
21		*Ancylolomia jacquelinae*	Rougeot, 1984	Arba Minch	E
22		*Ancylolomia shafferi*	Rougeot, 1977	Koffolé [Kofale]	E
23		*Ancylolomia shefferialis*	Rougeot, 1984	Bahar Dar	E
24		*Chilo luniferalis*	Hampson, 1896	Abyssinia [Ethiopia]	E
25		*Classeya aphrodite*	Błeszyński, 1964	Dire Dawa	E
26		*Crambus arnaudiae*	Rougeot, 1977	Koffolé [Kofale]	E
27		*Crambus bachi*	Bassi, 2012	Bahar Dar, Lake Tana	E
28		*Crambus bellinii*	Bassi, 2014	Bale Mts, Sanetti Plateau	
29		*Crambus boislamberti*	Rougeot, 1977	Dinsho Reserve	E
30		*Crambus dedalus*	Bassi, 2000	Karsan, Kollubi	E
31		*Crambus descarpentriesi*	(Rougeot, 1977)	Koffolé [Koffale]	E
32		*Crambus jupiter*	Błeszyński, 1963	Ethiopia SW, Gamu-Gofa, Konso	E
33		*Crambus netuncus*	Bassi, 2012	Near Debra Libanos	E
34		*Crambus richteri*	Błeszyński, 1963	Kaffa, Ghimira	E
35		*Dembea venulosella*	Ragonot, 1888	Abyssinia [Ethiopia]	
36		*Euchromius donum*	Schouten, 1988	Haro-Ali, Gurra	E
37		*Euclasta sidamona*	Rougeot, 1977	Koffolé [Koffale]	E
38		*Euctenospila castalis*	Warren, 1892	Abyssinia [Ethiopia]	
39		*Leucinodes ethiopica*	Mally, Korycinska, Agassiz, Hall, Hodgetts & Nuss, 2015	Dire Dawa Region, Dire Dawa District, Dire Dawa	
40		*Lygropia nigricornis*	Hampson, 1898	Abyssinia [Ethiopia]	
41		*Noorda trimaculalis*	Amsel, 1965	Ethiopia SW, Gammu-Gofa, Konso	E
42	Crambidae	*Noorda unipunctalis*	Amsel, 1963	Konso	E
43		*Pagyda pulvereiumbralis*	(Hampson, 1918)	Diré Daouá [Dire Dawa]	
44		*Pediasia ferruginea*	Błeszyński, 1963	Kaffa, Gembi	
45		*Pediasia simiensis*	Błeszyński, 1962	Soddu Province, Wolamo [Walita]	E
46		*Prionapteryx selenalis*	(Hampson, 1919)	Taddecha Mullka	E
47		*Prionotalis friesei*	Błeszyński, 1963	Ethiopia SW, Gamu-Gofa, Konso	E
48		*Tegostoma richteri*	Amsel, 1963	Awash	E
49	Depressariidae	*Odites aethiopicus*	Lvovsky, 2001	Kaffa, Gembi	
50	Elachistidae	*Elachista delocharis*	Meyrick, 1932	Jem-Jem Forest	E
51	Erebidae	*Achaea monodi*	Laporte, 1975	near Kebré-Mengist [Kibre Mengist]	E
52		*Afrasura rivulosa ethiopica*	Durante, 2009	Menegesha-Suba state Forest	E
53		*Afrasura indecisa orientalis*	Durante, 2009	Menegesha-Suba state Forest	E
54		*Afrasura terlinea*	Durante, 2009	Langano Lake	E
55		*Afrojavanica kostlani*	(Gaede, 1923)	Adis-Abeba	
56		*Alpenus geminipuncta*	(Hampson, 1916)	Abyssinia [Ethiopia]	E
57		*Amata alicia*	(Butler, 1876)	Abyssinia [Ethiopia]	
58		*Amata magrettii*	Berio, 1937	Metema [in Tigray, Ethiopia]	E
59		*Amata rufina*	(Oberthür, 1878)	Abyssinia [Ethiopia]	
60		*Amata shoa*	(Hampson, 1898)	Abyssinia [Ethiopia]	
61		*Amata velatipennis*	Walker, 1865	Marako	
62		*Amphicallia kostlani*	Strand, 1911	Gipfel des Sugyala	E
63		*Amsacta nigrisignata*	Gaede, 1923	Addis Ababa	E
64		*Amsactarctia radiosa*	(Pagenstecher, 1903)	Darassum	
65		*Anomis sabulifera*	(Guenée, 1852)	Abyssinia [Ethiopia]	
66		*Antiophlebia bourgognei*	Laporte, 1975	Arba Minch	E
67		*Aroa quadriplagata*	Pagenstecher, 1903	Galata	E
68		*Asura xanthophaea*	Toulgoët, 1977	Ethiopia	E
69		*Beriodesma smithii*	(Holland, 1897)	River Darde	
70		*Brunia birketsmithi*	(Toulgoët, 1977)	Kébré-Mengist [Kibre Mengist]	E
71		*Brunia dorsti*	(Toulgoët, 1977)	Kébré-Mengist [Kibre Mengist]	E
72		*Callophisma viettei*	Laporte, 1975	Arba Minch	E
73		*Carcinarctia rougeoti*	Toulgoët, 1977	Bale Reserve, Dinsho	E
74		*Casama impura*	(Hering, 1926)	Abyssinia [Ethiopia]	
75		*Cautatha abyssinia*	Hacker, Fiebig & Stadie, 2019	Reg. South Nations, Bonga Guesthouse	
76		*Cautatha bifasciata*	Hacker, Fiebig & Stadie, 2019	Reg. South Nations, road Shishinda-Bonga, 6 km, w Wushwush	E
77		*Cerocala confusa*	Warren, 1913	Abyssinia [Ethiopia]	E
78		*Clytie thibauti*	Laporte, 1991	Kibre Mengist	E
79		*Corgatha hyperxantha*	Hacker, Fiebig & Stadie, 2019	Reg. South Nations, Bonga Guesthouse	E
80		*Corgatha minutulana*	Hacker, 2019	Southern Prov., 6 km ENE Weyto, Segen river	
81		*Cortyta canescens septentrionalis*	Hacker, 2016	12 km W of Jinka, near border of Mago National Park	
82		*Crambiforma leucostrepta*	Hampson, 1926	Harrar	E
83		*Crypsotidia digitata*	Kühne, 2005	Harar	E
84		*Crypsotidia gigantea*	Kühne, 2005	Harar	
85		*Ctenusa curvilinea*	Hampson, 1913	Taddecha Mullka	
86		*Cyana abyssinica*	Karisch, 2003	Akaki River, Addis Ababa	E
87	Erebidae	*Cyana ethiopica*	Karisch, 2013	near Kebré-Mengist [Kibre Mengist]	E
88		*Dasychira grisea*	Pagenstecher, 1903	Bone	E
89		*Dasychira plesia*	Collenette, 1938	Abyssinia [Ethiopia]	
90		*Digama meridionalis deliae*	Berio, 1939	Adu-Abuna [in Tigray, Ethiopia]	E
91		*Donuctenusa fiorii*	Berio, 1940	Ogaden, Uarder [Warder]	E
92		*Enargeiosia elegans*	(Butler, 1877)	Atbara*	
93		*Eublemma accedens aethiopica*	Hacker, 2019	Ethiopia, 3 km N Turmi, Mango Camping Site	
94		*Eublemma aethiopiana*	Hacker, 2019	Jinka, Mago Nat. Park, Magoriverside	
95		*Eublemma baccatrix*	Hacker, 2019	Southern Prov., 2.6 km EE Wondo Genet	
96		*Eublemma collacteana*	Hacker, 2019	12 km W Jimma, border Mago Nat. Park	
97		*Eublemma costivinata*	Berio, 1945	Borana Nagelli [Borena Nagelle]	E
98		*Eublemma diredaoua*	Hacker, 2019	Dire Daoua, Abyssinia	E
99		*Eublemma ferruginata*	Hacker, 2019	20 ESE Sashamane, Wendo Genet	
100		*Eublemma heteropaura*	Hacker, 2019	Oromia, 7 km NW Yabelo	
101		*Eublemma joergmuelleri*	Hacker & Schrier, 2019	Ethiopia, Awash N.P., Headquarter	E
102		*Eublemma perturbata*	Hacker, 2019	Oromia prov., 6.5 km ne Shebe	
103		*Eublemma plectoversa*	Hacker, 2019	8 km N Turmi	
104		*Eublemma schreieri*	Hacker, 2019	Oromia, 1km W vill. Aluweya	
105		*Eublemma sidamonia*	Hacker, Fiebig & Stadie, 2019	Sidamo, Yabello, vic. 6km SO near Deritu village	E
106		*Eublemma siticulina*	Hacker, 2019	Dire Daoua, Abyssinia	E
107		*Eublemma uhlenhuthi*	Wiltshire, 1988	Abyssinia, Dire Daoua [dire-dawa]	
108		*Euproctis chrysophaea*	(Walker, 1865)	Abyssinia [Ethiopia]	
109		*Eyralpenus scioana*	(Oberthür, 1880)	Scioa [Shoa]	
110		*Galtara doriae*	(Oberthür, 1880)	Mahal Uonz, between Harrar and Addis Abeba [Awash River]	
111		*Kenyarctia melanogastra*	(Holland, 1897)	Gof [Gofa]	
112		*Hypena abyssinialis*	Guenée, 1854	Abyssinia [Ethiopia]	
113		*Hypena padelekorum*	Lödl, 1995	Djem-Djem [Jem Jem] Forest	E
114		*Hypena philippi*	Laporte, 1991	Arba Minch	E
115		*Hyposada zavattarii*	Berio, 1944	Gondaraba	
116		*Hypotacha fiorii*	Berio, 1943	Diredaua [Dire Dawa]	
117		*Hypotacha glaucata*	(Holland, 1897)	Sjeikh Husein [Shek Hussein]	
118		*Ischnarctia cinerea*	(Pagenstecher, 1903)	Gogoru	
119		*Laelia dabano*	Collenette, 1934	Dabano River	
120		*Lithacodia awassensis*	Berio, 1984	Awassa Lake	E
121		*Lithacodia persubtilis*	Berio, 1984	Kebré-Mengist	E
122		*Marcipa rougeoti*	Pelletier, 1975	Kebré-Mengist	E
123		*Metachrostis debivar*	(Berio, 1947)	Ogaden, Ualual [Walwal]	E
124		*Metachrostis phaeographa*	Hacker, 2011	12 km W of Jinka, border Mago National Park	
125		*Metarctia carmel*	Kiriakoff, 1957	SW Abyssinia [Ethiopia], Kambatta	E
126	Erebidae	*Metarctia gada*	Rougeot, 1977	Dinsho Reserve, Réserve de Balé	E
127		*Metarctia haematricha*	Hampson, 1905	Kutai Metha	
128		*Metarctia kumasina*	Strand, 1920	Zegi Tsana [Zegie Tana]	
129		*Metarctia negusi*	Kiriakoff, 1957	Abyssinia [Ethiopia]	E
130		*Metarctia noctis*	Druce, 1910	Diré Daouá [Dire Dawa]	E
131		*Metarctia saalfeldi*	Kiriakoff, 1960	Villagio	E
132		*Metarctia unicolor*	(Oberthür, 1880)	Oromo Country, Fin-Fekéré	
133		*Micralarctia punctulatum purus*	(Butler, 1878)	Abyssinia [Ethiopia]	
134		*Oediblemma peregrina*	Hacker, Fiebig & Stadie, 2019	Reg. South Nations, Sheiko Forest Road Teppi Mizan Teferi	E
135		*Ophiusa dianaris*	(Guenée, 1852)	Abyssinia [Ethiopia]	
136		*Pantydia dufayi*	Laporte, 1975	Near Koffolé [Koffalé]	
137		*Paramarbla abyssinica*	Collenette, 1956	Birbir, Joubdo [Yubdo]	E
138		*Paraonagylla zavattarii*	Berio, 1939	Neghelli [Nagelle]	E
139		*Pericyma schreieri*	Hacker, 2016	Gamu-Gofa Province, 3 km N of Turmi	
140		*Phytometra angensteini*	Hacker, 2019	Arba Minch	E
141		*Plecopterodes melliflua*	(Holland, 1897)	Sjeikh Husein [Shek Hussein]	
142		*Plecopterodes molybdena*	Berio, 1954	Gorgorà, Lake Tana	E
143		*Podomachla antinorii*	(Oberthür, 1880)	Mahal Uonz [Awash River]	
144		*Polymona rufifemur ellisoni*	Collenette, 1938	Abyssinia [Ethiopia]	
145		*Proluta ethiopica*	(Hacker, 2011)	Arba Minch Region, Omo Province, Gemu Gofa	
146		*Pseudomicrodes varia*	Berio, 1944	Elolo	
147		*Pteredoa atripalpia*	Hampson, 1910	Atbara River	
148		*Rhabdophera exarata*	(Mabille, 1890)	Abyssinia [Ethiopia]	
149		*Ruanda nuda*	(Holland, 1897)	River Darde	
150		*Seydelia geometrica*	(Oberthür, 1883)	Scioa [Shoa]	
151		*Spilosoma mediopunctata*	(Pagenstecher, 1903)	Arbarone	
152		*Spilosoma quadrimacula*	Toulgoët, 1977	Lalokéli	E
153		*Stenilema aurantiaca*	Hampson, 1909	Abyssinia [Ethiopia]	
154		*Stenilema hailesellassiei*	(Birket-Smith, 1965)	Addis Ababa, University College Campus	E
155		*Stracena aegrota*	Le Cerf, 1922	Harar	E
156		*Stracilla translucida*	(Oberthür, 1880)	Scioa [Shoa], Mahal Uonz	
157		*Syngatha eremita*	Hacker, Fiebig & Stadie, 2019	Reg. South Nations, Bonga Guesthouse	E
158		*Syngatha parascotoides*	Hacker, 2019	12 km W Jinka, border Mago National Park	
159		*Syngatha simplicicata*	Hacker, Fiebig & Stadie, 2019	Reg. South Nations, Sheiko Forest Road Teppi Mizan Teferi	E
160		*Tegiapa ambiguosa*	Hacker, Fiebig & Stadie, 2019	Reg. South Nations, road Shishinda-Bonga, 6 km W Wushwush	
161		*Tegiapa obliqua*	Hacker, Fiebig & Stadie, 2019	Sidamo, Yabello vic., 10km W road to Konso	
162		*Tegiapa schreieri*	Hacker, 2019	Oromia Prov., 6 km ESE Jimma	
163		*Teracotona abyssinica*	(Rothschild, 1933)	Central Abyssinia [Ethiopia], Maraco [Marako]	
164		*Teracotona neumanni*	Rothschild, 1933	SW Abyssinia [Ethiopia], Kambatta	E
165	Erebidae	*Teracotona postalbida*	(Gaede, 1926)	Abyssinia [Ethiopia]	E
166		*Teracotona clara rubiginea*	(Toulgoët, 1977)	Fisha Genet	E
167		*Teracotona seminigra*	(Hampson, 1905)	Zegi Tsana [Tana]	E
168		*Thyretes negus*	Oberthür, 1878	Abyssinia [Ethiopia]	
169		*Tigreana nathaliannae*	Laporte, 1991	Wollo, Ataye	E
170		*Tigreana sandrae*	Laporte, 1991	Wollo Ataye	E
171		*Trigonodes exportata*	Guenée, 1852	Abyssinia [Ethiopia]	
172		*Tytroca alabuensis alabuensis*	Wiltshire, 1970	Alabu	
173		*Tytroca balnearia mutabilis*	Hacker, 2016	15 km N of Arba Minch, 2 km after junction to Chencha	E
174		*Tytroca heterophysa*	Hacker, 2016	Omo Region, Gemu Gofa Province, Arba Minch	E
175		*Ulotrichopus phaeoleucus griseus*	Kühne, 2005	Addis Ababa	
176		*Utetheisa amhara*	Jordan, 1939	Abyssinia [Ethiopia]	
177		*Zekelita heteroleuca*	Hacker, 2016	Southern Province, 11.2 km W of Bonga	E
178		*Zekelita lehmanni magnificaria*	Hacker, 2016	10.5 km W of Weyto	E
179		*Zekelita nilotica*	Hacker, 2016	30 km SE of Bahir Dar, Tisisat above Blue Nile falls	E
180	Eupterotidae	*Phiala abyssinica*	Aurivillius, 1904	Zegi Tsana [Tana]	E
181		*Phiala bergeri*	Rougeot, 1975	Bale	E
182		*Rhodopteriana abyssinica*	(Rothschild, 1917)	Harrar [Harar]	
183		*Rhodopteriana sidamoensis*	Darge, 2013	Sidamo Province, near Mega	E
184	Euteliidae	*Eutelia favillatrix*	(Guenée, 1852)	Abyssinia [Ethiopia]	
185		*Stenosticta schreieri*	Hacker, 2010	3 km N Turmi, Mango Camping Site	E
186	Gelechiidae	*Aphanostola maxima*	Bidzilya & Mey, 2016	Lake Tana, Bahir Dar	E
187		*Chrysoesthia parilis*	(Vári, 1963)	Little Akaki River, near Addis Ababa	E
188		*Stegasta sattleri*	Bidzilya & Mey, 2011	Addis Ababa	
189		*Stomopteryx ochrosema*	Meyrick, 1932	Addis Alam [Alem], ca. 20 miles W. of Addis Ababa	E
190		*Chiasmia abyssinica*	Krüger, 2001	Harrar [Harar]	E
191		*Chiasmia procidata*	(Guenée, 1858)	Abyssinia [Ethiopia]	
192		*Chiasmia streniata*	(Guenée, 1858)	Abyssinia [Ethiopia]	
193		*Chiasmia trinotatula*	Krüger, 2001	Kabarutar, 56 miles W of Lake Tana	E
194		*Cleora oculata sidamo*	Herbulot, 1977	Kébré-Mengist [Kibre Mengist]	E
195		*Cleora pavlitzkiae etesiae*	Fletcher, 1967	Harar	
196		*Coenina dentataria*	Swinhoe, 1904	Abyssinia [Ethiopia]	
197		*Comibaena theodori*	Hausmann & Parisi, 2014	Kaffa Province, 10 km N of Bonga	E
198		*Drepanogynis nigerrima*	(Swinhoe, 1904)	Abyssinia [Ethiopia]	E
199		*Epigynopteryx flavedinaria*	(Guenée, 1857)	Abyssinia [Ethiopia]	
200		*Epigynopteryx rougeoti*	Herbulot, 1977	Dinsho Marshes	E
201		*Epigynopteryx scotti*	Fletcher, 1959	Ethiopia N, Simien, near Mindigabsa	E
202		*Erastria marginata*	(Swinhoe, 1904)	Abyssinia [Ethiopia]	E
203		*Eupithecia angulata*	Fletcher, 1951	Harar	E
204		*Eupithecia dinshoensis*	Herbulot, 1983	Dinsho Col	E
205		*Eupithecia incommoda*	Herbulot, 1983	Dinsho Reserve	E
206		*Eupithecia inquinata*	Fletcher, 1950	Lekamti [Naqamte]	
207	Geometridae	*Eupithecia ochralba*	Herbulot, 1983	Dinsho Reserve	E
208		*Eupithecia pseudoabbreviata*	Fletcher, 1951	Harar	E
209		*Eupithecia rougeoti*	Herbulot, 1983	Dinsho Reserve	E
210		*Eupithecia urbanata*	Fletcher, 1956	Harar	E
211		*Geodena brunneomarginata*	Karisch, 2003	Shoa, 50 km W of Adis Ababa	E
212		*Hemistola aetherea*	Debauche, 1937	Addis Ababa	E
213		*Henicovalva negus*	Krüger, 2017	Dire Daoua [Dawa]	E
214		*Heterostegane serrata*	(Fletcher, 1958)	Diré Daouá [Dire Dawa]	
215		*Hydrelia candace*	Prout, 1929	Addis Ababa	E
216		*Hypochrosis chiarinii*	(Oberthür, 1883)	Scioa [Shoa]	
217		*Idaea glomerata*	(Prout, 1937)	Abyssinia [Ethiopia]	E
218		*Lomographa indularia*	(Guenée, 1858)	Abyssinia [Ethiopia]	
219		*Mimoclystia pudicata cecchii*	(Oberthür, 1883)	Scioa [Shoa], Let-Marefia [Jet Marafia]	E
220		*Nothofidonia xenoleuca*	Prout, 1928	Wolisso, between Hauash [Awash] and Omo	E
221		*Odontopera briela*	(Debauche, 1937)	Mt Chillálo [Chilalo]	E
222		*Odontopera integraria*	Guenée, 1858	Abyssinia [Ethiopia]	
223		*Odontopera protecta*	Herbulot, 1983	Dinsho Reserve	E
224		*Omphacodes pulchrifimbria pulchritacta*	Prout, 1923	Abyssinia [Ethiopia] Central Moraqui [Marako]	
225		*Oreometra ras*	Herbulot, 1983	near Mount Batu	E
226		*Piercia zukwalensis*	Debauche, 1937	Mt Zukwala/Cuqala	
227		*Pingasa abyssiniaria*	(Guenée, 1858)	Harar	
228		*Platypepla bifida*	Herbulot, 1984	near Kébré-Mengist [Kibre Mengist]	E
229		*Platypepla uhlenhuthi*	Krüger, 2001	Diré Daouá [Dire Dawa]	E
230		*Prasinocyma aquamarina*	Hausmann, Sciarretta & Parisi, 2016	Bale Mts, 10 km S Rira	E
231		*Prasinocyma aetheraea*	(Debauche, 1937)	Addis Ababa	E
232		*Prasinocyma albivenata*	Herbulot, 1983	Dinsho Marsh	E
233		*Prasinocyma amharensis*	Hausmann, Sciarretta & Parisi, 2016	SW Debre Sina & Sembo, Umg. Debre Sina	E
234		*Prasinocyma angolica pseudopedicata*	Hausmann, Sciarretta & Parisi, 2016	7 km NW Yabello	E
235		*Prasinocyma angulifera*	Hausmann, Sciarretta & Parisi, 2016	southern Bale Mts, Harenna Forest	E
236		*Prasinocyma batesi distans*	Hausmann, Sciarretta & Parisi, 2016	Addis Ababa	E
237		*Prasinocyma baumgaertneri*	Hausmann, Sciarretta & Parisi, 2016	Harenna Forest	E
238		*Prasinocyma beryllaria*	Hausmann, Sciarretta & Parisi, 2016	13 km W Yabello Motel	E
239		*Prasinocyma bongaensis*	Hausmann, Sciarretta & Parisi, 2016	Bonga, 12 km E	E
240		*Prasinocyma discipuncta*	Hausmann, Sciaretta & Parisi, 2016	16 km SW Kibre Mengist	E
241		*Prasinocyma fallax*	Hausmann, Sciarretta & Parisi, 2016	SW. Debre Sina & Sembo, Umg. Debre Sina	E
242		*Prasinocyma fusca*	Hausmann, Sciarretta & Parisi, 2016	Harenna Forest	E
243		*Prasinocyma gajdacsi*	Prout, 1930	Adis Abeba [Addis Ababa]	E
244		*Prasinocyma gemmifera*	Hausmann, Sciaretta & Parisi, 2016	Wushwush, 7.4 km w	E
245	Geometridae	*Prasinocyma germinaria*	(Guenée, 1857)	Abyssinia [Ethiopia]	
246		*Prasinocyma getachewi*	Hausmann, Sciarretta & Parisi, 2016	Arba Minch	E
247		*Prasinocyma hailei*	Debauche, 1937	Addis Ababa	E
248		*Prasinocyma immaculata thiaucourti*	Herbulot, 1993	Debre Zeit	E
249		*Prasinocyma leveneorum*	Hausmann, Sciarretta & Parisi, 2016	Harenna Forest, Karcha clearing	E
250		*Prasinocyma lutulenta*	Hausmann, Sciarretta & Parisi, 2016	Arba Minch	E
251		*Prasinocyma magica*	Hausmann, Sciarretta & Parisi, 2016	Mago National park	E
252		*Prasinocyma monikae*	Hausmann, Sciarretta & Parisi, 2016	13 km W Yabello, Motel	E
253		*Prasinocyma pedicata aethiopica*	Hausmann, Sciarretta & Parisi, 2016	16 km SW Kibre Mengist	E
254		*Prasinocyma robusta*	Hausmann, Sciarretta & Parisi, 2016	13 km W Yabello, Motel	E
255		*Prasinocyma septentrionalis*	Hausmann, Sciarretta & Parisi, 2016	Arba Minch	E
256		*Prasinocyma shoa shoa*	Herbulot, 1993	Debre Zeit	E
257		*Prasinocyma shoa yabellensis*	Hausmann, Sciarretta & Parisi, 2016	13 km W Yabello, Motel	E
258		*Prasinocyma stefani*	Hausmann, Sciarretta & Parisi, 2016	Bonga, 12 km E	E
259		*Prasinocyma tranquilla*	Prout, 1917	NW of Harar, Diredaua [Dire Dawa]	E
260		*Prasinocyma trematerrai simienensis*	Hausmann, Sciarretta & Parisi, 2016	Semien Mountains, chennek Camp	E
261		*Prasinocyma trematerrai trematerrai*	Hausmann, Sciarretta & Parisi, 2016	Dinsho	E
262		*Problepsis fiebigi*	Stadie & Stadie, 2016	Omo Region, Province of Gemu Gofa, Arba Minch	E
263		*Problepsis neumanni*	Prout, 1932	Djiren, Djimma [Jimma]	E
264		*Problepsis sihvoneni*	Stadie & Stadie, 2016	Sidamo, 13 km W of Yabello, Motel	E
265		*Protosteira decolorata*	Herbulot, 1984	Semyen, Sankaber	E
266		*Rhodometra labdoides*	Herbulot, 1997	Choa [Shoa], Debré Zeit	E
267		*Rhodometra plectaria*	(Guenée, 1857)	Abyssinia [Ethiopia]	
268		*Rougeotiella pseudonoctua*	Herbulot, 1983	Kébré-Mengist [Kibre Mengist]	E
269		*Scopula erymna*	Prout, 1928	Gurra, Dagaje	E
270		*Scopula scotti*	Debauche, 1937	Addis Ababa	
271		*Scopula silonaria*	(Guenée, 1858)	Abyssinia [Ethiopia]	
272		*Scopula simplificata*	Prout, 1928	NE Africa, Ganale River	E
273		*Sesquialtera lonchota*	Prout, 1931	Diré Daouá [Dire Dawa], NW of Harrar	E
274		*Somatina pythiaria*	(Guenée, 1857)	Abyssinia [Ethiopia]	
275		*Tephronia aethiopica*	Herbulot, 1983	Shoa, Menagesha Forest	E
276		*Traminda neptunaria*	(Guenée, 1857)	Abyssinia [Ethiopia]	
277		*Trimetopia aetheraria*	Guenée, 1858	Abyssinia [Ethiopia]	E
278		*Xanthisthisa copta*	Herbulot, 1977	Boré Forest	E
279		*Xanthisthisa terna*	Herbulot, 1984	Shoa, Menagesha Forest	E
280		*Xanthorhoe abyssinica*	Herbulot, 1983	Chensha	E
281		*Xanthorhoe alta*	Debauche, 1937	Mt Chillálo, Albaso	E
282	Geometridae	*Xanthorhoe cadra*	(Debauche, 1937)	Mt Chillálo, from forest of Kosso-trees	E
283		*Xanthorhoe cuneosignata*	Debauche, 1937	Mt Chillálo, Albaso	E
284		*Xanthorhoe excelsissima*	Herbulot, 1977	Mt Batu	E
285		*Xenimpia sabae amarei*	Hausmann, 2006	Arba Minch, Region of Omo, Gemu Gofa,	E
286		*Xylopteryx emunctaria*	(Guenée, 1858)	Abyssinia [Ethiopia]	
287		*Xylopteryx gada*	Herbulot, 2000	Balé, Harena Forest	E
288		*Xylopteryx raphaelaria*	(Oberthür, 1880)	Scioa [Shoa]	E
289		*Zamarada excavata pollex*	Fletcher, 1974	Jlubador [Ilubabor] Gore	E
290		*Zamarada hyalinaria*	(Guenée, 1857)	Abyssinia [Ethiopia]	
291		*Zamarada melasma*	Fletcher, 1974	Dire Daoua [Dire Dawa]	
292		*Zamarada secutaria*	(Guenée, 1857)	Abyssinia [Ethiopia]	
293		*Zamarada shoa*	Herbulot, 2002	Shoa, 50 km W of Addis Ababa	E
294		*Zamarada torrida*	Fletcher, 1974	Dire Daoua [Dire Dawa]	
295	Glyphipterigidae	*Ussara semicoronis*	Meyrick, 1932	Jem-Jem Forest	E
296	Gracillariidae	*Acrocercops heteroloba*	Meyrick, 1932	Jem-Jem Forest	E
297		*Acrocercops orianassa*	Meyrick, 1932	Mt Zukwala/Cuqala	
298		*Caloptilia macropleura*	(Meyrick, 1932)	Jem Jem Forest	
299		*Metacercops hexactis*	(Meyrick, 1932)	Jem-Jem Forest	E
300		*Metriochroa carissae*	Vári, 1963	Addis Ababa, Little Akaki River	E
301		*Metriochroa scotinopa*	Vári, 1963	Dabra Zeit [Debre Zeit]	E
302	Gracillariidae	*Porphyrosela homotropha*	Vári, 1963	Addis Ababa, Little Akaki River	E
303		*Stomphastis heringi*	Vári, 1963	Near Addis Ababa, Little Akaki River	E
304		*Stomphastis horrens*	(Meyrick, 1932)	Jem-Jem Forest	E
305	Hesperiidae	*Abantis meneliki*	Berger, 1979	Harrar	
306		*Apallaga menageshae*	Libert, 2014	Mt Menagesha, NW Addis Abeba	
307		*Coeliades chalybe immaculata*	Carpenter, 1935	Alanga River	E
308		*Coeliades menelik menelik*	(Ungemach, 1932)	Lilmo, dans la pays de Sayo	
309		*Eretis mixta*	Evans, 1937	Dire Daouna [Dire Dawa]	
310		*Metisella formosus mittoni*	Carcasson, 1961	Mega	E
311		*Sarangesa lucidella helena*	Evans, 1947	Harar	E
312	Lasiocampidae	*Beralade perobliqua monostrigata*	Berio, 1940	Adi-Abuna [in Tigray, Ethiopia]	E
313		*Bombycopsis abyssinica*	Joannou & Krüger, 2009	Addis Abeba	E
314		*Mallocampa toulgoeti*	Rougeot, 1977	Kébré-Mengist [Kibre Mengist]	E
315		*Odontocheilopteryx eothina*	Tams, 1931	Djoubdo [Yubdo], Birbir	E
316		*Odontocheilopteryx lajonquieri*	Rougeot, 1977	near Kébré-Mengist [Kibre Mengist]	E
317		*Pallastica hararia*	Zolotuhin & Gurkovich, 2009	Harar	E
318		*Sena donaldsoni rougeoti*	Lajonquière, 1977	Arba Minch	E
319		*Sena scotti*	(Tams, 1931)	Djem-Djem [Jem-Jem] Forest	
320		*Stoermeriana abbayensis*	(Rougeot, 1984)	Bahar-Dar, marais du Nil Bleu, Abbay	E
321		*Stoermeriana chavailloni*	(Rougeot, 1984)	Melka-Kontouré	E
322		*Stoermeriana das*	(Hering, 1928)	Eli	E
323		*Stoermeriana laportei*	(Rougeot, 1977)	Kébré-Mengist [Kibre Mengist]	E
324		*Stoermeriana murinuscolor*	(Rougeot, 1984)	Shoa, Menagesha Forest	E
325		*Stoermeriana saanayetae*	(Rougeot, 1984)	Awassa Lake	E
326		*Stoermeriana tamsi*	(Rougeot, 1977)	Dinsho Marshes, Balé	E
327		*Stoermeriana viettei*	(Rougeot, 1977)	Dinsho Marshes	E
328	Limacodidae	*Crothaema flava*	Berio, 1940	Adi-Abuna [in Tigray, Ethiopia]	E
329		*Hamartia johanni*	Rougeot, 1977	Kébré-Mengist [Kibre Mengist]	E
330		*Hamartia medora moulini*	Rougeot, 1977	Kébré-Mengist [Kibre Mengist]	E
331		*Jordaniana lactea*	(Pagenstecher, 1903)	Ganale	
332	Lycaenidae	*Anthene amarah*	(Guérin-Méneville, 1849)	Dire Dawa	
333		*Anthene butleri butleri*	(Oberthür, 1880)	Mantek; Mahal-Uonz	
334		*Anthene chojnackii*	Libert, 2010	10 km NW of Neghelli	E
335		*Anthene confusa*	Libert, 2010	Touloudimtou [Tullu Dimtu]	
336		*Anthene contrastata*	(Ungemach, 1932)	Bedelle	E
337		*Anthene definita nigrocaudata*	(Pagenstecher, 1902)	Ginir	E
338		*Anthene dulcis*	(Pagenstecher, 1902)	Gambe beim Abasse-See	
339		*Anthene hodsoni*	(Talbot, 1935)	Kibish River	
340		*Anthene opalina janna*	Gabriel, 1949	Fich-Babile Road	
341		*Anthene opalina opalina*	Stempffer, 1946	Callafo[Kalafo], Webi Shebeli, Ogaden	
342		*Anthene pitmani aethiopana*	Libert, 2010	Ghibe River, Addis Abeba-Jimma road	
343		*Anthene princeps*	(Butler, 1876)	Atbara*	
344		*Anthene saddacus*	(Talbot, 1935)	Ethiopia	E
345		*Anthene suquala*	(Pagenstecher, 1902)	Suquala	
346		*Axiocerses maureli*	Dufrane, 1954	Harrar	E
347		*Azanus jesous*	(Guérin-Méneville, 1849)	Abyssinie [Ethiopia]	
348		*Cacyreus ethiopicus*	(Tite, 1961)	25 km north of Quiha	E
349		*Cacyreus fracta ghimirra*	Talbot, 1935	Shoa Ghimirra province	E
350		*Chilades elicola*	(Strand, 1911)	Eli, Ethiopia	
351		*Deudorix lorisona baronica*	Ungemach, 1932	Baro River	E
352		*Deudorix ungemachi*	Libert, 2004	Ethiopia	E
353		*Eicochrysops antoto*	(Strand, 1911)	Umgebung unterhalb Antotos [Entoto]	E
354		*Eicochrysops meryamae*	Rougeot, 1983	Province de Gondar, environs de Debarek	E
355		*Eicochrysops messapus sebagadis*	(Guérin-Méneville, 1849)	Abyssinie [Ethiopia]	
356		*Euchrysops abyssinicus*	(Aurivillius, 1922)	Tchafianani; Debasso	E
357		*Euchrysops cyclopteris*	(Butler, 1876)	Atbara*	
358		*Euchrysops mauensis Abyssinia [Ethiopia]e*	Storace, 1950	Bahrdàr [Bahar Dar] sulle rive meridionali del Lago Tana	E
359		*Euchrysops nandensis*	(Neave, 1904)	Lake Tana	
360		*Hypolycaena ogadenensis*	Stempffer, 1946	Dagahbur, Ogaden	E
361		*Iolaus crawshayi maureli*	Dufrane, 1954	Harrar [Harar]	
362		*Iolaus piaggiae*	Oberthür, 1883	Kolla di Giagaguè-Agher	E
363		*Lachnocnema abyssinica*	Libert, 1996	Dire Daouna [Dawa]	
364		*Lepidochrysops abyssiniensis abyssiniensis*	(Strand, 1911)	Eli	E
365		*Lepidochrysops abyssiniensis oculus*	(Ungemach, 1932)	Ouama	E
366		*Lepidochrysops guichardi*	Gabriel, 1949	10 miles West of Addis Ababa	E
367		*Lepidochrysops lunulifer*	(Ungemach, 1932)	Didessa	E
368		*Lepidochrysops negus*	(Felder & Felder, 1865)	Africa septentrionali-orientalis: Bogo	
369		*Lepidochrysops pterou lilacina*	(Ungemach, 1932)	Didessa	E
370	Lycaenidae	*Lepidochrysops subvariegata*	Talbot, 1935	Dirre Dawa	E
371		*Leptomyrina boschi*	Strand, 1911	Abyssinie [Ethiopia]n	E
372		*Lycaena phlaeas pseudophlaeas*	(Lucas, 1866)	Abyssinie [Ethiopia]	E
373		*Myrina silenus nzoiae*	Stoneham, 1937	Western Kenya to Ethiopia and Eritrea	
374		*Pentila pauli ras*	Talbot, 1935	S.W. Abyssinia [Ethiopia], Pokodi [Bokoji]	E
375		*Stugeta bowkeri ethiopica*	(Stempffer & Bennett, 1958)	Harrar [Harar]	E
376		*Tarucus ungemachi*	Stempffer, 1942	Rivière Baro Abyssinie [Ethiopia] occidentale	
377		*Thermoniphas colorata*	(Ungemach, 1932)	Youbdo	
378		*Tuxentius cretosus*	(Butler, 1876)	Atbara*	
379		*Tuxentius kaffana*	(Talbot, 1935)	Nado’s Province, Yeki; Mocha District, Gamadura	E
380		*Uranothauma antinorii*	(Oberthür, 1883)	Torrente di Sciotalit	
381		*Uranothauma nubifer distinctesignatus*	(Strand, 1911)	[Ethiopia]	E
382		*Zintha hintza resplendens*	(Butler, 1876)	Atbara*	E
383	Metarbelidae	*Aethiopina semicirculata*	Gaede, 1929	Abyssinia [Ethiopia]	E
384		*Salagena fetlaworkae*	Rougeot, 1977	near Koffolé [Koffale]	E
385		*Teragra lemairei*	Rougeot, 1977	Dinsho Marches	E
386		*Teragra villiersi*	Rougeot, 1977	near Koffolé [Koffale]	E
387	Noctuidae	*Abrostola obliqua*	Dufay, 1958	Abyssinia [Ethiopia]	E
388		*Abrostola rougeoti*	Rougeot, 1977	near Koffolé [Koffale]	E
389		*Acontia albatrigona*	Hacker, Legrain & Fibiger, 2008	Arba Minch Region, Omo, Province Gemu, Gofa	
390		*Acontia amarei*	Hacker, Legrain & Fibiger, 2010	Gamu-Gofa Province, 10.5 km W of Weyto	E
391		*Acontia amhara*	Hacker, Legrain & Fibiger, 2008	Gamu-Gofa Province, 8 km N of Turmi	E
392		*Acontia proesei*	Hacker, Legrain & Fibiger, 2008	Valley of the river Tekezé, 30 km N of Gashena	E
393		*Acontia robertbecki*	Hacker, Legrain & Fibiger, 2010	Arba Minch Region, Gemu Gofa Province	E
394		*Acontia ruficincta*	Hampson, 1910	Atbara*	E
395		*Acontia secta*	Guenée, 1852	Abyssinia [Ethiopia]	
396		*Acontia uhlenhuthi*	Hacker, Legrain & Fibiger, 2008	Diré Daouá [Dire Dawa]	E
397		*Acontiola boursini*	(Berio, 1940)	Lekemti [Naqamte]	
398		*Acrapex abbayei*	Laporte, 1984	Dinsho Reserve	E
399		*Acrapex apexangula*	Laporte, 1984	near Koffolé [Koffale]	E
400		*Acrapex ausseili*	Laporte, 1984	Fisha Genet	E
401		*Acrapex franeyae*	Laporte, 1984	Dinsho Reserve	E
402		*Acrapex genrei*	Laporte, 1984	Dinsho Reserve	E
403		*Acrapex girardi*	Laporte, 1984	Dinsho Reserve	E
404		*Acrapex guiffrayorum*	Laporte, 1984	Dinsho Reserve	E
405		*Acrapex mastawatae*	Laporte, 1984	Arba Minch	E
406		*Acrapex matilei*	Laporte, 1984	Dinsho Reserve	E
407		*Acrapex satanas*	Laporte, 1984	Dinsho Reserve	E
408		*Acrapex soyema*	Le Ru, 2017	Gibe, Soyema Bridge	E
409		*Acrapex ulmii*	Laporte, 1991	Koffole [Koffale]	E
410		*Acrapex zaouditou*	Laporte, 1991	Koffole [Koffale]	E
411		*Aedia albirena*	(Hampson, 1926)	Taddecha Mullha	
412	Noctuidae	*Aedia konsonata*	Hacker, 2016	Konso	E
413		*Aedia marmoreata*	Hacker, 2016	12 km W of Jinka	
414		*Aegocera ferrugo*	Jordan, 1926	Hora Daka	E
415		*Agrotis baleense*	Laporte, 1977	Dinsho, Bale Reserve	
416		*Agrotis cinchonina*	Guenée, 1852	Abyssinia [Ethiopia]	E
417		*Agrotis debivari*	(Berio, 1962)	Africa Orientale Italiana, Debivar	E
418		*Agrotis separata*	Guenée, 1852	Abyssinia [Ethiopia]	E
419		*Amazonides berioi*	(Laporte, 1984)	Lekemti [Naqamte]	E
420		*Amazonides berliozi*	Laporte, 1974	Dinsho Col	E
421		*Amazonides dubiomeodes*	Laporte, 1977	Kébré-Mengist [Kibre Mengist]	E
422		*Amazonides ezanai*	(Laporte, 1984)	Kébré-Mengist [Kibre Mengist]	E
423		*Amazonides fumigera*	Laporte, 1977	Dinsho Marshes	E
424		*Amazonides koffoleense*	Laporte, 1977	Koffolé [Koffale]	E
425		*Amazonides laheuderiae*	Laporte, 1984	Abba Hoye-Gara	E
426		*Amazonides pseudoberliozi*	Rougeot & Laporte, 1983	Simyen, Sankaber	E
427		*Amazonides putrefacta*	(Guenée, 1852)	Abyssinia [Ethiopia]	
428		*Amazonides ungemachi*	(Laporte, 1984)	Ioubdo, Birbir, Nole Kabe	E
429		*Amazonides zarajakobi*	Laporte, 1984	Dinsho Marshes	E
430		*Amphia hepialoides*	Guenée, 1852	Abyssinia [Ethiopia]antio	E
431		*Aporophoba subaustralis*	Berio, 1977	Addis Ababa	E
432		*Apospasta albirenalis*	Laporte, 1974	Mt Batu	E
433		*Apospasta diffusa*	Laporte, 1974	Dinsho Col	E
434		*Apospasta erici*	Laporte, 1984	Dinsho Reserve	E
435		*Apospasta incongrua*	Laporte, 1974	Dinsho Col	E
436		*Apospasta maryamae*	Laporte, 1974	Dinsho Marshes	E
437		*Apospasta niger*	Laporte, 1974	Dinsho Marshes	E
438		*Apospasta rougeoti*	Laporte, 1991	Boré Forest	
439		*Apospasta rufa*	Laporte, 1991	Choa [Shoa], Menageshah [Menegasha] Forest	E
440		*Apospasta sabulosa*	Fletcher, 1959	Simien, Lori	E
441		*Apospasta thomasi*	Laporte, 1991	Addis Ababa	E
442		*Ariathisa abyssinia*	(Guenée, 1852)	Abyssinia [Ethiopia]	
443		*Aspidifrontia ungemachi*	(Laporte, 1978)	Metti	E
444		*Athetis aeschrioides*	Berio, 1940	Adi-Abuni [in Tigray, Ethiopia]	
445		*Athetis carayoni*	Laporte, 1977	Dinsho col	E
446		*Athetis viettei*	Laporte, 1991	Choa [Shoa], Melka-Kontoure [Melka Konture]	E
447		*Axylia aregashae*	Laporte, 1984	near Kébré-Mengist [Kibre Mengist]	E
448		*Axylia bryi*	Laporte, 1984	Dinsho Marshes	E
449		*Axylia destefanii*	Berio, 1944	El-Dire	
450		*Axylia gabriellae*	Laporte, 1975	Boré Forest	E
451		*Axylia marthae*	Laporte, 1984	near Koffolé [Koffale]	E
452		*Axylia orbicularis*	Laporte, 1984	near Kébré-Mengist [Kibre Mengist]	E
453		*Axylia sanyetiensis*	Laporte, 1984	near Mt Batu	E
454		*Axylia vespertina*	Laporte, 1984	near Kébré-Mengist [Kibre Mengist]	E
455		*Batuana abbahoyegarana*	Rougeot, 1983	Abba Hoye-Gara, Wollo	E
456		*Batuana exspectata*	Laporte & Rougeot, 1981	Gojam, Mt Choke	E
457		*Batuana lobeliarum*	Laporte, 1976	near Dinsho	E
458		*Batuana rougeoti*	Laporte, 1976	near Mt Batu	E
459	Noctuidae	*Berionycta beckroberti*	Kiss, 2017	15 km E of Yabello	E
460		*Berionycta behouneki*	Kiss, 2017	13 km W of Yabello	E
461		*Berionycta berioi*	Kiss, 2017	12 km NNE of Arba Minch	E
462		*Berionycta nigra*	Kiss, 2017	15 km E of Yabello	E
463		*Berionycta orbicularis*	Kiss, 2017	15 km E of Yabello	E
464		*Berionycta ponticamima*	Kiss, 2017	15 km E of Yabello	E
465		*Capillamentum gelleyi*	Laporte, 1984	Addis Ababa	E
466		*Caradrina atriluna*	Guenée, 1852	Abyssinia [Ethiopia]	
467		*Caradrina torpens*	Guenée, 1852	Abyssinia [Ethiopia]	E
468		*Carcharoda erlangeri*	Rothschild, 1924	Waute Merehan [Mreham]	E
469		*Cirrodes rosaceus*	Rothschild, 1924	Waute Merehan [Mreham]	E
470		*Claudaxylia dinshoense*	Laporte, 1984	Dinsho Reserve	E
471		*Compsotata corneliae*	Behounek & Beck, 2012	Bale Mountains, Province of Bale, Hangasso	E
472		*Conservula ludocaroli*	(Laporte, 1991)	Debre Zeit	E
473		*Conservula scriptura*	(Rougeot & Laporte, 1983)	Simyen, Sankaber	E
474		*Cucullia simoneaui*	Laporte, 1976	Bale Reserve, Dinsho	E
475		*Cucullia tedjicolora*	Laporte, 1977	Kébré-Mengist [Kibre Mengist]	E
476		*Eucladodes achrorophilus*	Laporte, 1976	Near Mt Batu	E
477		*Eucladodes baleensis*	Laporte, 1976	Bale Reserve	E
478		*Euplexia imperator*	Laporte, 1984	Dinsho Marshes	E
479		*Euplexia mercieri*	Laporte, 1984	Arussi, near Koffolé [Koffale]	E
480		*Euplexia pinoni*	Laporte, 1984	Kébré-Mengist [Kibre Mengist]	E
481		*Euplexia shoana*	Laporte, 1984	Shoa, near Hosana	E
482		*Euxoa dodolaense*	Laporte, 1984	Road to Dodola	E
483		*Euxoa montigenarum*	Rougeot & Laporte, 1983	Simyen, Sankaber	E
484		*Euxoa semyenensis*	Laporte, 1991	Sankaber	E
485		*Euxoa waliarum*	Rougeot & Laporte, 1983	Simyen, Sankaber	E
486		*Feliniopsis duponti*	Laporte, 1974	near Kebré-Mengist [Kibre Mengist]	
487		*Feliniopsis germainae*	Laporte, 1975	near Kebré-Mengist [Kibre Mengist]	E
488		*Feliniopsis insolita*	Hacker & Fibiger, 2007	Addis Ababa, Sholla	E
489		*Feliniopsis jinka*	Hacker, 2010	Gamu-Gofa Province, 10 km W of Jinka	
490		*Feraxinia jemjemensis*	(Laporte, 1984)	Kébré-Mengist [Kibre Mengist]	E
491		*Heliophobus africana*	Berio, 1977	Addis Ababa	E
492		*Heliothis saskai*	(Berio, 1975)	Addis Ababa	E
493		*Hemituerta mahdi*	(Pagenstecher, 1903)	Hanadscho [Dinsho district]	
494		*Heraclia viettei*	Kiriakoff, 1973	Nole Kaba	E
495		*Hermonassoides abyssinica*	(Berio, 1975)	Addis Ababa	E
496		*Hermonassoides dinshoensis*	(Laporte, 1977)	Dinsho Marshes	E
497		*Hermonassoides marmorata*	(Laporte, 1977)	Fisha Genet	E
498		*Hermonassoides mauricei*	(Laporte, 1975)	Koffale	
499		*Hermonassoides mendeboense*	(Laporte, 1984)	Dinsho	E
500		*Hermonassoides minosi*	(Laporte, 1991)	Managesha Forest	E
501		*Hermonassoides scipioni*	(Laporte, 1977)	Dinsho, Bale Reserve	E
502		*Hiccoda clarae*	Berio, 1947	Ogaden, Uarder [Warder]	E
503		*Hyperfrontia direae*	Berio, 1962	Dire-Daoua [Dire Dawa]	E
504		*Hyperfrontia limbata*	Berio, 1962	El-Dire	E
505		*Koffoleania michaellae*	Laporte, 1977	near Koffolé [Koffale]	E
506		*Leucania aedesiusi*	Rougeot & Laporte, 1983	Simyen, Sankaber	E
507		*Leucania argyrina*	Laporte, 1984	Bahar Dar	E
508	Noctuidae	*Leucania claudicans*	Guenée, 1852	Abyssinia [Ethiopia]	E
509		*Leucania cyprium*	(Laporte, 1984)	Dinsho Marshes	E
510		*Leucania fasilidasi*	(Laporte, 1984)	Dinsho Marshes	E
511		*Leumicamia oreias*	(Fletcher, 1959)	Simien, above Lori	E
512		*Leumicamia palustris*	Laporte, 1976	Dinsho Marshes	E
513		*Leumicamia venustissima*	(Laporte, 1974)	Bale Reserve	E
514		*Lophotarsia girmai*	Laporte, 1975	Arba Minch	E
515		*Lophotarsia leucoplagoides*	(Berio, 1941)	El-Dire	E
516		*Lophotarsia theresae*	Beck & Behounek, 2013	Bale Mountains National Park, region Oromia/Sidamo, Province of Bale, 4 km W of Sura	E
517		*Maghadena ingridae*	Laporte, 1977	Dinsho Reserve, Balé	E
518		*Maliattha eburnea*	Hacker, 2016	Oroma Province, 6 km ESE of Jimma	
519		*Matopo berhanoui*	Laporte, 1984	Melka-Kontouré [Konture]	E
520		*Mentaxya bruneli*	Laporte, 1975	near Kebré-Mengist [Kibre Mengist]	E
521		*Mentaxya fouqueae*	Laporte, 1974	Boré Forest	
522		*Mentaxya inconstans*	Laporte, 1984	Dinsho Marshes	E
523		*Mentaxya lacteifrons*	Laporte, 1984	Kébré-Mengist [Kibre Mengist]	E
524		*Michelliana afroalpina*	Laporte, 1976	near Mt Batu	E
525		*Micraxylia antemedialis*	Laporte, 1975	near Kebré-Mengist [Kibre Mengist]	E
526		*Micraxylia hypericoides*	Berio, 1962	Oromo e Sidamo, Neghelli [Neghelle]	E
527		*Micraxylia lividoradiata*	(Berio, 1940)	Adi-Abuna [in Tigray, Ethiopia]	E
528		*Mythimna altiphila*	Hreblay & Legrain, 1996	Addis Abeba [Ababa]	
529		*Mythimna amlaki*	Laporte, 1984	Near Mt Batu	E
530		*Mythimna bisetulata*	(Berio, 1940)	Adi-Abuna [in Tigray, Ethiopia]	E
531		*Mythimna germanae*	Laporte, 1991	Melka Kontouré	E
532		*Neostichtis teruworkae*	Laporte, 1984	Near Hosana	E
533		*Nocthadena griseoviridis*	Laporte, 1976	Near Mt Batu	E
534		*Numeniastes selenis*	Fletcher, 1963	Harar	
535		*Nyodes biardi*	Laporte, 1984	Shashemane	E
536		*Ochropleura sidamona*	Laporte, 1977	Fisha-Genet	
537		*Odontestra richinii*	Berio, 1940	Adi-Abuna [in Tigray, Ethiopia]	E
538		*Odontestra variegata*	Berio, 1940	Adi-Abuna [in Tigray, Ethiopia]	
539		*Odontestra vitta*	Berio, 1975	Addis Ababa	E
540		*Oligia adactricula*	Guenée, 1852	Abyssinie [Ethiopia]	E
541		*Oligia arbaminchensis*	Laporte, 1991	Arba Minch	E
542		*Oligia genettae*	Laporte, 1991	Kebre-Mengist [Kibre Mengist]	E
543		*Omphalestra nellyae*	(Berio, 1939)	Adua [in Ethiopia]	E
544		*Ozarba alberti phaeoxantha*	Hacker, 2016	Dire Dawa	
545		*Ozarba didymochra*	Hacker, 2016	Gamu-Gofa Province, 8 km E of Weyto	E
546		*Ozarba fuscundosa*	Hacker, 2016	Oromia, 3 km NNE of Finchawa	
547		*Ozarba grisescens*	Berio, 1947	Harrar [Harar], Dire Daua [Dire Dawa]	
548		*Ozarba latizonata*	Hacker, 2016	Gamu-Gofa Province, 8 km E of Weyto	
549		*Ozarba naumanni*	Hacker, 2016	Gamo Gofa Province, Konso	E
550	Noctuidae	*Ozarba permutata*	Hacker, 2016	20 km ESE of Sashemene, Wondo Genet	
551		*Ozarba rubrofusca*	Berio, 1947	Ogaden, Uarder [Werder]	E
552		*Ozarba tenuis*	Hacker, 2016	Province of Gamo Gofa, 8 km N of Turmi	E
553		*Ozarba uhlenhuthi*	Hacker, 2016	Dire Dawa	E
554		*Phyllophila corgatha*	Berio, 1984	Arba Minch	E
555		*Phyllophila richinii*	Berio, 1940	Adi-Abuna [in Tigray, Ethiopia]	E
556		*Pseudozarba nilotica*	Hacker, 2016	30 km SE of Bahir Dar, Tisisat above Blue Nile Falls	E
557		*Pusillathetis fiorii*	Berio, 1976	Uarder [Warder in Ogaden]	
558		*Ramesodes oblonga*	Berio, 1976	Adi Abuna [in Tigray, Ethiopia]	
559		*Rhodochlaena dinshoense*	Laporte, 1974	Dinsho Marshes	E
560		*Rougeotia abyssinica*	(Hampson, 1918)	Kutai Mecha	E
561		*Rougeotia aethiopica*	Laporte, 1974	Dinsho swamp	E
562		*Rougeotia ludovici*	Laporte, 1974	Bale Reserve	E
563		*Rougeotia ludovicoides*	Laporte, 1977	Dinsho Marshes	E
564		*Rougeotia obscura*	Laporte, 1974	Dinsho Col	E
565		*Rougeotia roseogrisea*	Laporte, 1974	Near Mt Batu	E
566		*Rougeotia rougeoti*	Laporte, 1984	Mt Batu Forest	E
567		*Schinia ennatae*	(Laporte, 1984)	Addis Ababa	E
568		*Schinia magdalenae*	(Laporte, 1976)	Bale Reserve, Dinsho	E
569		*Schinia ungemachi*	(Berio, 1945)	Oromo Sidamo, Uollega [Wollega]	E
570		*Schinia xanthiata*	(Berio, 1940)	Adi-Abuna [in Tigray, Ethiopia]	E
571		*Sciomesa boulardi*	(Laporte, 1984)	near Koffolé [Koffale]	E
572		*Sciomesa excelsa*	(Laporte, 1976)	Near Mt Batu	E
573		*Sciomesa franciscae*	Laporte, 1991	Choa, Hosana	E
574		*Sciomesa secata*	Berio, 1977	Addis Ababa	E
575		*Sesamia enanouae*	Laporte, 1991	Gojam, Bahr-Dar, marais du Nil Bleu	E
576		*Sesamia roumeti*	Laporte, 1991	Gojam, Bahr-Dar	E
577		*Solgaitiana petrosi*	Laporte, 1984	Kébré-Mengist [Kibre Mengist]	E
578		*Spodoptera excelsa*	Rougeot & Laporte, 1983	Simyen, Sankaber	E
579		*Subnoctua arbaminchensis*	Laporte, 1984	Arba Minch	E
580		*Thiacidas robertbecki*	Hacker & Zilli, 2007	Awassa, Awassa Lake, Bale Region	E
581		*Tholeropsis decimata*	Berio, 1977	Addis Ababa	E
582		*Tholeropsis uncinata*	Berio, 1977	Addis Ababa	E
583		*Thysanoplusia asapheia*	(Dufay, 1977)	near Koffolé [Koffale]	
584		*Thysanoplusia dolera*	Dufay, 1977	near Koffolé [Koffale]	E
585		*Timora flavocarnea*	Hampson, 1903	Abyssinia [Ethiopia]	
586		*Timora zavattarii*	Berio, 1944	El-Dire	E
587		*Tracheplexia annabellae*	Laporte, 1991	Menagesha Forest	E
588		*Tracheplexia colettae*	Laporte, 1991	Gemu-Gofa, Arba-Minch	E
589		*Tracheplexia leguerni*	Laporte, 1984	Fort Wosha	E
590		*Tracheplexia petryvesi*	Laporte, 1991	Menagesha Forest	E
591		*Tracheplexia richinii*	Berio, 1973	Adiu Abuna [in Tigray, Ethiopia]	
592		*Tycomarptes adami*	Laporte, 1974	Dinsho Col	E
593		*Tycomarptes aethiopica*	Laporte, 1974	Mt Batu	E
594		*Tycomarptes berioi*	Laporte, 1974	Boré Forest	E
595		*Tycomarptes bipuncta*	Laporte, 1974	Boré Forest	E
596		*Tycomarptes bipunctatoides*	Laporte, 1974	near Koffolé [Koffale]	E
597	Noctuidae	*Tycomarptes gelladarum*	Rougeot & Laporte, 1983	Simyen, Sankaber	E
598		*Tycomarptes inferior*	(Guenée, 1852)	Abyssinia [Ethiopia]	
599		*Tycomarptes journiaci*	Laporte, 1977	Near Mt Batu	E
600		*Tycomarptes limoni*	Laporte, 1974	near Koffolé [Koffale]	E
601		*Tycomarptes semyensis*	Rougeot & Laporte, 1983	Simyen, Sankaber	E
602		*Tycomarptes thibauti*	Laporte, 1974	Boré Forest	E
603		*Vietteania chojnackii*	(Laporte, 1984)	Dinsho Marshes	E
604	Nolidae	*Arcyophora zanderi*	Felder & Rogenhofer, 1875	Abyssinia [Ethiopia]	
605		*Bryophilopsis martinae*	Laporte, 1991	Gemu-Gofa, Konso	E
606		*Characoma adiabunensis*	Berio, 1940	Adi-Abuna [in Tigray, Ethiopia]	E
607		*Earias richinii*	Berio, 1940	Adi-Abuni [in Tigray, Ethiopia]	
608		*Eligma neumanni*	Rothschild, 1924	Blue Nile, Abera Koritscha, Uata Dera	E
609		*Escarpamenta damarana abyssinica*	Hacker, 2013	6 km E of Weyto, Weyto River	E
610		*Evonima littoralis abyssinica*	Hacker, 2012	Southern Province, Jinka, Mago National Park, 350 m SW of Headquarter,	E
611		*Gigantoceras villiersi*	Laporte, 1975	Arba Minch	E
612		*Meganola cerographa*	Hacker, 2012	Oromia District, 6.5 km N of Bonga	E
613		*Meganola coffeana*	Hacker, 2012	Oromia Province, 6.5 km NE of Shebe	
614		*Meganola ethiopica*	Hacker, 2012	Addis Ababa	E
615		*Meganola harenna*	Hacker, 2014	Harenna Forest, Karcha Camp Ground	E
616		*Meganola leucometabola*	Hacker, 2012	Oromia Province, 6.5 km N of Bonga	
617		*Meganola longisigna*	Hacker, 2012	Oromia Region, 1km W. of village Aluweya	
618		*Meganola lupii*	Hacker & Hausmann, 2012	Oromia Province, 13 km S. of Agere Maryam	E
619		*Meganola pachygrapha*	Hacker, 2012	Oromia Province, 6.5 km N of Bonga	
620		*Meganola poliovittata*	Hacker, 2012	Oromia Province, 6 km ESE of Jimma	E
621		*Meganola pyrrhomorpha*	Hacker, 2012	Oromia Province, 6.5 km N of Bonga	E
622		*Meganola simillima*	Hacker, 2012	Oromia District, 13 km S of Agere Maryam	E
623		*Meganola stadiensis*	Hacker, 2014	Harenna Forest, Karcha Camp Ground	E
624		*Meganola stigmatolalis*	Hacker, 2012	Southern Province, 23 km WSW of Welkite, Gibe River	
625		*Meganola unilineata*	Hacker, 2012	Southern Province, 11.2 km W of Bonga	E
626		*Neaxestis mesogonia*	Hampson, 1905	Atbara R.	
627		*Nola abyssinica*	Hacker, 2012	Oromia Province, 13 km S of Agere Maryam	
628		*Nola afrotaeniata*	Hacker, 2012	12 km W Jinka, border Mago National Park	
629		*Nola amhara*	Hacker, 2012	Addis Ababa	
630		*Nola angensteini*	Hacker, 2012	Afar Region, NE of Mile Serdo Wildlife Refuge, Tendaho	
631	Nolidae	*Nola balealpina*	Hacker, 2012	Oromia Province, Bale Mountains National Park, Disho	E
632		*Nola calochromata*	Hacker, 2014	Harenna Forest, Harenna Forest Road	E
633		*Nola destituta*	Hacker, 2012	Oromia Province, 8 km W of Nazret	E
634		*Nola jarzabekae*	Hacker, 2012	Oromia Province, Abiyata-Shala-Hayak National Park	E
635		*Nola omphalota euroetes*	Hacker, 2012	Oromia Province, 6 km ESE of Jimma	
636		*Nola socotrensis vansoni*	Hacker, 2012	12 km W Jinka, border Mago NP	
637		*Nola sphaeromorpha*	Hacker, 2012	Oromia Province, 13 km S of Agere Maryam,	E
638		*Nolidia platygrapha*	Hacker, 2012	Amhara Region, W of Mirab, Gojam Zone, 15 km NW of Bahar Dar	E
639	Notodontidae	*Afroplitis quadratus*	(Viette, 1954)	River Baro	E
640		*Antheua birbirana*	Viette, 1954	middle course of Birbir, Youbdo	E
641		*Antheua gaedei*	Kiriakoff, 1962	Addis Ababa	E
642		*Antheua trivitta*	(Hampson, 1910)	Abyssinia [Ethiopia]	E
643		*Antistaura decorata*	Kiriakoff, 1965	Derdaua, North-East of Harrar	E
644		*Boscawenia nora*	(Pagenstecher, 1903)	Ganale	E
645		*Desmeocraera kiriakoffi*	Thiaucourt, 1977	near Kébré-Mengist [Kibre Mengist]	E
646		*Eutimia smithii*	Holland, 1897	Dombalok	E
647		*Polelassothys callista abyssinica*	Viette, 1954	Moy. Dedissa [Didessa]	E
648		*Psalisodes saalfeldi*	Kiriakoff, 1979	Al Abed	E
649		*Scalmicauda azebae*	Thiaucourt, 1977	near Kébré-Mengist [Kibre Mengist]	E
650		*Thaumetopoea apologetica abyssinica*	Strand, 1911	Addis Ababa	
651		*Tricholoba rougeoti*	Thiaucourt, 1977	Arba Minch	E
652	Nymphalidae	*Acraea aganice orientalis*	(Ungemach, 1932)	Bouré	
653		*Acraea alcinoe nado*	(Ungemach, 1932)	Bouré	E
654		*Acraea chilo chilo*	Godman, 1880	Kalamet, Sebka Valley	
655		*Acraea doubledayi*	Guérin-Méneville, 1849	Abyssinie [Ethiopia]	
656		*Acraea epaea homochroa*	(Rothschild & Jordan, 1905)	Banka, Malo	E
657		*Acraea kakana*	Eltringham, 1911	Adie Kaka, Kafa	E
658		*Acraea oscari*	Rothschild, 1902	Banka, Malo	E
659		*Acraea poggei ras*	(Ungemach, 1932)	Oullaga [Wollega]	E
660		*Acraea zetes sidamona*	Rothschild & Jordan, 1905	Alata, Sidamo	E
661		*Acraea zoumi*	Pierre, 1995	Ethiopia	E
662		*Amauris echeria steckeri*	Kheil, 1890	Abessynia	
663		*Amauris hecate stictica*	Rothschild & Jordan, 1903	Anderatscha	E
664		*Amauris niavius aethiops*	Rothschild & Jordan, 1903	Anderatscha	
665		*Amauris ochlea darius*	Rothschild & Jordan, 1903	Anderatscha	
666		*Antanartia abyssinica*	(C. & R. Felder, [1867])	Ethiopia	E
667		*Antanartia schaeneia diluta*	Rothschild & Jordan, 1903	Kaffa	E
668		*Argynnis hyperbius neumanni*	Rothschild, 1902	Kaffa	E
669		*Aterica galene incisa*	Rothschild & Jordan, 1903	between Kankati and Djibbe, Djimma [Jimma]	E
670	Nymphalidae	*Bicyclus pavonis*	(Butler, 1876)	Abyssinia [Ethiopia]	
671		*Bicyclus safitza aethiops*	(Rothschild & Jordan, 1905)	Lake Abassi	E
672		*Charaxes etesipe abyssinicus*	Rothschild, 1900	Sciotalit, Sxioa [Shoa]	E
673		*Charaxes eurinome birbirica*	(Ungemach, 1932)	Youbdo	
674		*Charaxes figini*	van Someren, 1969	Eritaea, Setit, El Eghin [Ethiopia]	
675		*Charaxes galawadiwosi*	Plantrou & Rougeot, 1979	Arba-Minch	E
676		*Charaxes hansali hansali*	Van Someren, 1971	Africa septentrionali-orientalis: Bogos	
677		*Charaxes jahlusa ganalensis*	Carpenter, 1937	Salakle, Ganale river”	
678		*Charaxes junius junius*	Oberthür, 1883	Scioa [Shoa]	E
679		*Charaxes junius somalicus*	Rothschild, 1900	Harrar Highlands, Somaliland	
680		*Charaxes kirki daria*	Rothschild, 1903	Jabalo	E
681		*Charaxes lactetinctus ungemachi*	Le Cerf, 1927	Youbdo (Birir)	
682		*Charaxes larseni*	Rydon, 1982	Jambo area, Nanji Hill	E
683		*Charaxes numenes neumanni*	Rothschild, 1902	Wori to Gamitscha, Kaffa	E
684		*Charaxes pelias pagenstecheri*	Poulton, 1926	S Ethiopia	
685		*Charaxes phoebus*	Butler, 1866	Abyssinia [Ethiopia]	E
686		*Charaxes rectans*	Rothschild & Jordan, 1903	Upper Urga, Kollu, Schoa [Shoa]	
687		*Charaxes saturnus pagenstecheri*	Poulton, 1926	S. Abyssinia [Ethiopia]	E
688		*Charaxes sidamo*	Plantrou & Rougeot, 1979	Kébré-Mengist [Kibre Mengist]	E
689		*Charaxes tiridates marginatus*	Rothschild & Jordan, 1903	Scheko	E
690		*Eronia cleodora cleodora*	Hübner, [1823]	Ethiopia	
691		*Eronia leda*	(Boisduval, 1847)	Marako	
692		*Euphaedra caerulescens submarginalis*	Hecq, 1997	[Ethiopia?]	E
693		*Euphaedra castanoides deficiens*	Hecq, 1997	West, Didessa River	E
694		*Euphaedra medon abouna*	Ungemach, 1932	Youbdo	E
695		*Euphaedra neumanni*	Rothschild, 1902	Scheko [Sheko]	
696		*Euphaedra sarita abyssinica*	Rothschild, 1902	Kankati forest, Djimma	E
697		*Eurytela hiarbas abyssinica*	Rothschild & Jordan, 1903	Banka	E
698		*Euxanthe eurinome birbirica*	Ungemach, 1932	Youbdo	
699		*Hypolimnas salmacis platydema*	Rothschild & Jordan, 1903	Scheko	E
700		*Junonia terea fumata*	(Rothschild & Jordan, 1903)	Gillet Mountains	
701		*Lasiommata maderakal*	(Guérin-Méneville, 1849)	Abyssinie [Ethiopia]	
702		*Melitaea abyssinica*	Oberthür, 1909	Abyssinie [Ethiopia]	E
703		*Neptis nemetes obtusa*	Rothschild & Jordan, 1903	Scheko	E
704		*Phalanta eurytis microps*	(Rothschild & Jordan, 1903)	Walenso [Woliso], Gillet Mts	E
705		*Phalanta phalantha aethiopica*	(Rothschild & Jordan, 1903)	Gillet Mts	
706		*Pseudacraea boisduvalii sayonis*	Ungemach, 1932	Oumbi	E
707		*Pseudacraea eurytus mimoras*	Ungemach, 1932	Oumbi	E
708		*Pseudacraea lucretia walensensis*	(Sharpe, 1896)	Waenso [Woliso]	E
709		*Sevenia boisduvali kaffana*	(Rothschild & Jordan, 1903)	Godjeb to Bonga, Kaffa	E
710	Nymphalidae	*Telchinia aurivillii schecana*	Rothschild & Jordan, 1905	Scheko [Sheiko]	E
711		*Telchinia bonasia banka*	Eltringham, 1912	Banka, Malo	
712		*Telchinia guichardi*	Gabriel, 1949	Lekempti	E
713		*Telchinia jodutta aethiops*	Rothschild & Jordan, 1905	Dereta Mts	E
714		*Telchinia necoda*	Hewitson, 1861	Abyssinia [Ethiopia]	E
715		*Telchinia peneleos gelonica*	(Rothschild & Jordan, 1905)	Upper Gelo River	E
716		*Telchinia perenna kaffana*	(Rothschild, 1902)	Kaffa	E
717		*Telchinia pharsalus rhodina*	Rothschild, 1902	Kaffa	E
718		*Telchinia rangatana maji*	Carpenter, 1935	Maji Province	E
719		*Telchinia safie antinorii*	(Oberthür, 1880)	Mahal-Uonz	E
720		*Telchinia safie safie*	(C. & R. Felder, 1865)	Abyssinia [Ethiopia] Meridionalis	E
721		*Telchinia ungemachi*	(Le Cerf, 1927)	Youbdo (Birbi)	E
722		*Tirumala formosa neumanni*	(Rothschild & Jordan, 1903)	Kaffa	E
723		*Vanessa abyssinica abyssinica*	Vane-Wright & Hughes, 2007	Ethiopia	
724		*Ypthima impura paupera*	Ungemach, 1932	Soubé-Boro	
725		*Ypthima simplicia*	Butler, 1876	Atbara*	
726	Papilionidae	*Graphium almansor birbiri*	(Ungemach, 1932)	Baro	E
727		*Graphium angolanus baronis*	(Ungemach, 1932)	Baro	
728		*Papilio arnoldiana*	Vane-Wright, 1995	S.W. Abyssinia [Ethiopia], Grine	E
729		*Papilio dardanus antinorii*	Oberthür, 1883	Abissinia, Feleklek and Sciotalit	E
730		*Papilio echerioides leucospilus*	Rothschild, 1902	Gara Mulata near Harar”	E
731		*Papilio echerioides oscari*	Rothschild, 1902	Kaffa and Djima [Jimma]	E
732		*Papilio microps*	Storace, 1951	Shoa, Abyssinia [Ethiopia] centrale	
733		*Papilio nireus pseudonireus*	Felder & Felder, 1865	Africa Septentrionali Oriental, Bogos	
734		*Papilio rex abyssinicana*	Vane-wright, 1995	S. W. Abyssinia [Ethiopia], Ganji River	E
735		*Papilio wilsoni*	Rothschild, 1926	Nubar Hills, Taldi	E
736	Pieridae	*Appias sylvia abyssinica*	Talbot, 1932	Joubda (Birbir)	E
737		*Belenois gidica abyssinica*	(Lucas, 1852)	Abyssinie [Ethiopia]	E
738		*Belenois gidica hypoxantha*	(Ungemach, 1932)	Gambela	E
739		*Belenois raffrayi*	(Oberthür, 1878)	Lac de Tzana [Lake Tana]	
740		*Belenois subeida hailo*	(Ungemach, 1932)	Nolé Kaba [in Wollega]	E
741		*Belenois thysa tricolor*	Talbot, 1943	Abyssinia [Ethiopia]	
742		*Belenois zochalia gada*	(Ungemach, 1932)	Nole-Kaba [in Wollega]	E
743		*Colias electo meneliki*	Berger, 1940	Gondar	
744		*Colias erate marnoana*	Rogenhofer, 1884	Ethiopia	
745		*Colotis antevippe zera*	(Lucas, 1852)	Abyssinie [Ethiopia]	
746		*Colotis celimene celimene*	(Lucas, 1852)	Abyssinie [Ethiopia]	
747		*Colotis danae eupompe*	(Klug, 1829)	in Arabia deserta, in Sinai monte, in Dongala et Habessinia	
748		*Colotis euippe exole*	(Reiche, 1850)	Abyssinie [Ethiopia]	
749		*Colotis hetaera aspasia*	(Ungemach, 1932)	Baro	
750		*Colotis phisadia ocellatus*	(Butler, 1886)	Somali-land [False locality]	E
751		*Colotis ungemachi*	(Le Cerf, 1922)	N Ethiopia	E
752		*Dixeia charina septentrionalis*	(Bernardi, 1958)	Djemdjem	E
753	Pieridae	*Eronia leda pupillata*	Strand, 1911	Marako	E
754		*Euchloe belemia abyssinica*	Riley, 1928	Mt. Chillalo	E
755		*Eurema desjardinsii regularis*	(Butler, 1876)	Atbara*	
756		*Leptosia alcesta pseudonuptilla*	Bernardi, 1959	Haute-Orguessa	
757		*Mylothris erlangeri*	Pagenstecher, 1902	Gewidscha	E
758		*Mylothris mortoni balkis*	Ungemach, 1932	Alenga	E
759		*Mylothris mortoni mortoni*	Blachier, 1912	Kaffa, dans l’Abyssinie [Ethiopia] meridionale”	E
760		*Mylothris rueppellii*	(Koch, 1865)	Abessynica	
761		*Mylothris sagala swaynei*	Butler, 1899	Harar Highlands	E
762		*Mylothris yulei amhara*	Ungemach, 1932	Alenga	E
763		*Pieris brassicoides*	Guérin-Méneville, 1849	Abyssinie [Ethiopia]	
764		*Pontia daplidice aethiops*	(De Joannis & Verity, 1913)	Abyssinie [Ethiopia]	E
765	Plutellidae	*Lepocnemis metapelista*	Meyrick, 1932	Jem-Jem Forest	E
766		*Plutella dryoxyla*	Meyrick, 1932	Mt Chillálo	E
767		*Plutella oxylopha*	Meyrick, 1932	Mt Chillálo	E
768		*Plutella stichocentra*	Meyrick, 1932	Mt Chillálo	E
769	Psychidae	*Acanthopsyche chrysora*	Bourgogne, 1980	Arba Minch	E
770		*Oiketicoides aethiopica*	Bourgogne, 1991	Wollo, Lalibela	E
771		*Taleporia aethiopica*	Strand, 1911	Mahenge	E
772	Pterophoridae	*Cosmoclostis gorbunovi*	Ustjuzhanin & Kovtunovich, 2011	West Shewa, 2 km S of Ambo	E
773		*Hellinsia aethiopicus*	(Amsel, 1963)	Gembi	
774		*Hellinsia ambo*	Ustjuzhanin & Kovtunovich, 2011	West Shewa, 2 km S of Ambo	
775		*Hellinsia bigoti*	(Rougeot, 1983)	Simyen, Sankaber	E
776		*Hellinsia negus*	(Gibeaux, 1994)	Wondo-Genet	E
777		*Merrifieldia lonnvei*	Gielis, 2011	Amhara Region, S of Debub, Gondar zone, 8 km NW of Addis Zemen, Highway 3	E
778		*Paracapperia esuriens*	Meyrick, 1932	Jem Jem Forest	
779		*Platyptilia daemonica*	Meyrick, 1932	Jem Jem Forest	E
780		*Platyptilia gondarensis*	Gibeaux, 1994	Gondar Province	
781		*Platyptilia implacata*	Meyrick, 1932	Jem Jem Forest	E
782		*Pterophorus lindneri*	(Amsel, 1963)	Gore	E
783		*Stenoptilia aethiopica*	Gibeaux, 1994	Sidamo, Wondo-Genet	
784		*Stenoptilia amharae*	Gielis, 2011	Amhara Region, Semien North, Gondar zone, 17 km NEE of Debark, Simien Mts National Park	E
785		*Stenoptilia rougeoti*	Gibeaux, 1994	Bale, marais de Dinsho	E
786		*Stenoptilia tyropiesta*	Meyrick, 1932	Mt Chillálo	E
787	Pyralidae	*Aglossodes dureti*	(Rougeot, 1977)	Arba Minch	E
788		*Aglossodes navattae*	Rougeot, 1977	Arba Minch	E
789		*Bostra excelsa*	Rougeot, 1984	near Mt Batu	E
790		*Bostra pseudoexcelsa*	Rougeot, 1984	Arba Minch	E
791		*Dembea venulosella*	Ragonot, 1888	Abyssinia [Ethiopia]	
792		*Ematheudes pollex*	Shaffer, 1998	Kosogay Wagra	E
793		*Emmalocera erythrinella*	(Ragonot, 1888)	Abyssinia [Ethiopia]	
794		*Endotricha ellisoni*	Whalley, 1963	Harar	
795		*Harraria rufipicta*	Hampson, 1930	Harrar [Harar]	E
796		*Loryma albilinealis*	Hampson, 1917	Diré Daouá [Dire Dawa]	E
797	Pyralidae	*Megarthridia christyi*	Rougeot, 1984	Arba Minch	E
798		*Nussia rougeoti*	Leraut, 2015	Koffolé [Koffale]	
799	Saturniidae	*Aurivillius cadioui*	Bouyer, 2008	100 kn E of Addis Ababa	E
800		*Bunaeopsis birbiri*	Bouvier, 1929	Joubdo (Birbir)	E
801		*Bunaeopsis oubie*	(Guérin-Méneville, 1849)	Abyssinia [Ethiopia]	
802		*Eosia digennaroi*	Bouyer, 2008	Bale, S of Omar	
803		*Epiphora antinorii*	(Oberthür, 1880)	Scioa [Shoa], Mahal Uonz [Awash River]	
804		*Epiphora bauhiniae atbarina*	(Butler, 1877)	Atbara*	
805		*Epiphora fourneri*	Rougeot, 1974	Road Koffolé-Arussi [Koffale- Arsi]	
806		*Gonimbrasia belina abayana*	Rougeot, 1977	Arba Minch	E
807		*Gonimbrasia belina felderi*	Rothschild, 1895	Bogos	E
808		*Gonimbrasia ellisoni*	Lemaire, 1962	Harar	E
809		*Gonimbrasia fletcheri*	Rougeot, 1960	Ethiopia	E
810		*Gonimbrasia fucata*	Rougeot, 1978	Ethiopia	E
811		*Goodia smithii*	(Holland, 1897)	East Africa [Ethiopia]	E
812		*Gynanisa arba*	Darge, 2008	Arba Minch	E
813		*Heniocha digennaroi*	Bouyer, 2008	Sidamo, Neguele Borana	E
814		*Holocerina digennariana*	Darge, 2008	Shashemene (Arsi)	E
815		*Ludia hansali*	Felder, 1874	Bogos	
816		*Ludia pupillata*	Strand, 1911	Antottos	E
817		*Micragone leonardi*	Bouyer, 2008	Sidamo, Dilla	E
818		*Nudaurelia fasciata*	Gaede, 1927	[Ethiopia]	E
819		*Nudaurelia ungemachti*	Bouvier, 1926	Djemdejm [Jem Jem]	E
820		*Pseudobunaea heyeri citrinarius*	Gaede, 1927	Harrar [Harar]	
821		*Pseudobunaea megana*	Darge, 2012	Sidamo Province, near Mega	E
822		*Urota melichari*	Bouyer, 2008	Sidamo Province, 15 km S of Negele	E
823	Scythrididae	*Scythris ethiopica*	Bengtsson, 2014	Lake Tana, Bahir Dar	E
824	Sesiidae	*Agriomelissa aethiopica*	(Le Cerf, 1917)	Abyssinia [Ethiopia]	E
825		*Jerbeia darkovi*	Gorbunov, 2018	Oromia, 21.8 km NW (289.5°) of Dembi Dolo	E
826		*Melittia abyssiniensis*	Hampson, 1919	Harar	E
827		*Melittia ambo*	Gorbunov, 2015	West Shewa, 3 km S of Ambo	E
828	Sphingidae	*Ceridia heuglini*	(Felder C. & Felder R., 1874)	Abyssinia [Ethiopia]	
829		*Ceridia quirini*	Sulak, Naumann & Witt, 2016	Oromia Region, road between Deritu and Dubuluk, near Deritu	E
830		*Chaerocina ellisoni*	Hayes, 1963	Harar	E
831		*Covelliana berioi*	Eitschberger & Melichar, 2016	near Debark Gondar	E
832		*Covelliana robertbecki*	Eitschberger & Melichar, 2016	Ethiopia Central, Oromia, southern Bale Mts, Harenna Forest	E
833		*Dovania dargei*	Pierre, 2000	Metu	E
834		*Dovania neumanni*	Jordan, 1926	SW Abyssinia [Ethiopia], Dhimma [Jimma]	E
835		*Falcatula tamsi*	Carcasson, 1968	Harrar [Harar]	E
836		*Leucophlebia neumanni*	Rothschild, 1902	Gelo River to Akobo River	
837		*Lophostethus dumolinii riedeli*	Eitschberger & Ströhle, 2011	Arba Minch	E
838	Sphingidae	*Lophostethus negus*	Jordan, 1926	SW Abyssinia [Ethiopia], Kambatta	E
839		*Macropoliana chrismonika*	Eitschberger & Melichar, 2016	Ethiopia W, 12 km E of Bonga	E
840		*Macropoliana haileselassiei*	Eitschberger & Melichar, 2016	Sidamo Province, 20 km S of Angere Maryam	E
841		*Macropoliana kingstoni*	Eitschberger, 2016	Oromia Region, 25 km E of Bonga/Mera	E
842		*Macropoliana stroehlei*	Eitschberger, 2016	Near Dorze	E
843		*Nephele xylina*	Rothschild & Jordan, 1910	Abyssinia [Ethiopia]	
844		*Platysphinx dorsti*	Rougeot, 1977	Kébré-Mengist [Kibre Mengist]	E
845		*Praedora melichari*	Haxaire, 2011	Sidamo Province, near Bitata	E
846		*Pseudoclanis bianchii*	(Oberthür, 1883)	Scioa [Shoa]	E
847		*Rufoclanis numosae rostislavi*	Haxaire & Melichar, 2008	Gamo Gofa Province, Dagabule National Park	E
848		*Temnora arida*	Melichar & Řezáč & Ilčíková, 2016	Dorze, Guge Mts	
849		*Temnora robusta*	Melichar, Řezáč & Ilčíková, 2016	Kaffa Prov., 40 km SW Jima,	
850		*Theretra ankae*	Melichar & Řezáč, 2015	Asosa	E
851	Thyrididae	*Arniocera cyanoxantha*	(Mabille, 1893)	Abyssinia [Ethiopia]	
852		*Arniocera guttulosa*	Jordan, 1915	Harar	E
853		*Lamprochrysa amata*	(Druce, 1910)	Diré Daouá [Dire Dawa]	E
854	Tineidae	*Afrocelestis minuta*	(Gozmány, 1965)	Gamu-Gofa, Konso	
855		*Ateliotum convicta*	(Meyrick, 1932)	Jem Jem Forest	E
856		*Ceratophaga luridula*	(Meyrick, 1932)	Mt Chillálo, moorland	E
857		*Ceratophaga nephelotorna*	(Meyrick, 1932)	Jem-Jem Forest	E
858		*Criticonoma spinulosa*	Gozmány, 1965	Gamu-Gofa, Konso	E
859		*Crypsithyris stenovalva*	(Gozmány, 1965)	Gamu-Gofa, Konso	E
860		*Dryadaula glycinoma*	(Meyrick, 1932)	Jem-Jem Forest	E
861		*Ectabola pygmina*	(Gozmány, 1965)	Marako	
862		*Edosa torrifacta*	(Gozmány, 1965)	Harrar [Harar]	E
863		*Hapsifera gypsophaea*	Gozmány, 1965	Gamu-Gofa, Konso	E
864		*Hapsifera pachypsaltis*	Gozmány, 1965	Kaffa, Ghimira	
865		*Hapsifera richteri*	Gozmány, 1965	Ethiopia SW, Gamu-Gofa, Konso	E
866		*Leptozancla zelotica*	(Meyrick, 1932)	Jem-Jem Forest	E
867		*Monopis addenda*	Gozmány, 1965	Kaffa, Gembi [Gimbi]	
868		*Monopis leopardina*	Gozmány, 1965	Kaffa, Abaro	E
869		*Monopis triplacopa*	Meyrick, 1932	Jem-Jem Forest, 45 miles W. of Addis-Ababa,	E
870		*Perissomastix lucifer*	Gozmány, 1965	Muger Valley	E
871		*Scalmatica separata*	Gozmány, 1965	Konso, Gamu-Gofa	E
872		*Silosca mariae*	Gozmány, 1965	Djerrer Valley	
873		*Tinissa spaniastra*	Meyrick, 1932	Jem-Jem Forest, , 45 miles from Addis Ababa	
874	Tortricidae	*Acleris baleina*	Razowski & Trematerra, 2010	Bale Mountains, Sanetti Plateau	E
875		*Acleris harenna*	Razowski & Trematerra, 2010	Bale Mountains, Harenna Forest, Karcha Camp	E
876		*Ancylis colaccii*	Razowski & Trematerra, 2012	Wellega Zone, Didessa River	E
877		*Bubonoxena alatheta*	Razowski & Trematerra, 2010	Bale Mountains, Harenna Forest, Karcha Camp	E
878		*Choristoneura palladinoi*	Razowski & Trematerra, 2010	Bale Mountains, Harenna Forest	E
879		*Coccothera carolae*	Razowski & Trematerra, 2010	Bale Mountains, Harenna Forest	
880	Tortricidae	*Coccothera triorbis*	Razowski & Trematerra, 2010	Bale Mountains, Harenna Forest	E
881		*Coniostola separata*	Razowski & Trematerra, 2010	Bale Mountains, Harenna Forest, Karcha Camp	E
882		*Cosmetra anepenthes*	(Razowski & Trematerra, 2010)	Bale Mountains, Harenna Forest, Karcha Camp	E
883		*Cosmetra latiloba*	(Razowski & Trematerra, 2010)	Bale Mountains, Harenna Forest, Karcha Camp	E
884		*Cydia calliglypta*	(Meyrick, 1932)	Jem-Jem Forest, edge of forest	E
885		*Cydia dinshoi*	Razowski & Trematerra, 2010	Bale Mountains, Dinsho Lodge	E
886		*Cydia lathetica*	Razowski & Trematerra, 2010	Bale Mountains, Dinsho Lodge	E
887		*Cydia tytthaspis*	Razowski & Trematerra, 2010	Bale Mountains, Harenna Forest, Karcha Camp	E
888		*Eccopsis aegidia*	(Meyrick, 1932)	Jem-Jem Forest	
889		*Eccopsis brunneopostica*	Razowski & Trematerra, 2010	Bale Mountains, Harenna Forest, Karcha Camp	E
890		*Eccopsis maschalista*	(Meyrick, 1932)	Jem-Jem Forest	E
891		*Eccopsis subincana*	Razowski & Trematerra, 2010	Bale Mountains, Harenna Forest	E
892		*Endothenia albapex*	(Razowski & Trematerra, 2010)	Bale Mountains, Harenna Forest	E
893		*Endothenia ethiopica*	Razowski & Trematerra, 2010	Bale Mountains, Harenna Forest, Karcha Camp	E
894		*Epichoristodes spilonoma*	(Meyrick, 1932)	Jem Jem Forest	
895		*Eucosma vulpecularis*	Meyrick, 1932	Jem-Jem Forest	E
896		*Eucosmocydia zegieana*	Razowski & Trematerra, 2018	Amhara, Zegie Peninsula	E
897		*Grapholita insperata*	Razowski & Trematerra, 2010	Bale Mountains, Dinsho Lodge	E
898		*Gypsonoma giorgiae*	Razowski & Trematerra, 2012	Ilubabor zone, Bedelle, Dabeda River	E
899		*Lozotaenia karchana*	Razowski & Trematerra, 2010	Bale Mountains, Harenna Forest, Karcha Camp	E
900		*Lozotaenia sciarrettae*	Razowski & Trematerra, 2010	Bale Mountains, Harenna Forest, Karcha Camp	E
901		*Megaherpystis oromiae*	Razowski & Trematerra, 2018	Oromia, Suba Forest	E
902		*Megaherpystis subae*	Razowski & Trematerra, 2018	Oromia, Suba Forest	E
903		*Megalota lygaria*	Razowski & Trematerra, 2010	Bale Mountains, Harenna Forest	E
904		*Metamesia physetopa*	(Meyrick, 1932)	Jem-Jem Forest and Mt Chillálo	
905		*Multiquaestia aequivoca*	Razowski & Trematerra, 2010	Bale Mountains, Harenna Forest	E
906		*Olethreutes didessae*	Razowski & Trematerra, 2012	Wellega zone, Didessa River	E
907		*Olethreutes polymorpha*	(Meyrick, 1932)	Jem-Jem Forest	E
908		*Parabactra addisalema*	Razowski & Trematerra, 2018	Oromia, Addis Alem, Ambo Park	E
909		*Paraeccopsis addis*	Aarvik, 2014	Addis Ababa	E
910		*Phtheochroa lonnvei*	Aarvik, 2010	Oromia Province, Bale zone, 43 km SW of Goba, Bale Mts National Park, Darwin Camp	E
911		*Plutographa xanthala*	Razowski & Trematerra, 2010	Bale Mountains, Dinsho Lodge	E
912		*Procrica dinshona*	Razowski & Trematerra, 2010	Bale Mountains, Dinsho Lodge	
913		*Procrica ophiograpta*	(Meyrick, 1932)	em-Jem Forest and Mt Chillálo	
914		*Procrica parisii*	Razowski & Trematerra, 2010	Bale Mountains, Dinsho Lodge	E
915		*Russograptis albulata*	Razowski & Trematerra, 2010	Bale Mountains, Harenna Forest	E
916		*Thaumatographa amarana*	Razowski & Trematerra, 2018	Amhara, Zegie Peninsula	E
917		*Thaumatovalva spinai*	Razowski & Trematerra, 2010	Omo Valley, Dowro Zone, Tarcha	
918		*Tortrix diametrica*	Meyrick, 1932	Jem-Jem Forest	E
919		*Trachybyrsis chionochlaena*	Meyrick, 1932	Mt Chillálo	E
920	Uraniidae	*Arussiana herbuloti*	Rougeot, 1977	near Koffolé [Koffale]	
921	Yponomeutidae	*Yponomeuta ocypora*	(Meyrick, 1932)	Jem-Jem Forest	E
922		*Yponomeuta ioni*	Agassiz, 2019		E
923		*Yponomeuta ocypora*	Meyrick, 1932		
924		*Yponomeuta oromiensis*	Agazzia, 2019		E
925	Zygaenidae	*Alteramenelikia jordani*	(Alberti, 1954)	Abyssinia [Ethiopia]	
926		*Astyloneura bicoloria*	Röber, 1929	Abyssinia [Ethiopia]	E
927		*Epiorna abessynica*	(Koch, 1865)	Abyssinia [Ethiopia]	
928		*Saliunca anhyalina*	Alberti, 1957	Abyssinia [Ethiopia]	E
929		*Saliunca homochroa*	(Holland, 1897)	Darde River	

Given these numbers, knowledge on the Ethiopian butterflies and moths appear to be particularly unsatisfactory, when compared to their (estimated) potential total numbers with other countries. For instance, the two most diverse European Mediterranean countries, i.e., France and Italy, with a combined land surface comparable to Ethiopia, have ca. 5,109 and 5,086 species of Lepidoptera, respectively (Stoch 2003; Wikipedia 2011).

To better evaluate the level of knowledge of the lepidopteran fauna in Ethiopia, and to roughly estimate the real biodiversity, we can compare it with neighboring Kenya, which for several aspects can be considered similar to Ethiopia, but probably it has been better investigated. So far, from Kenya approximately 4,815 lepidopteran taxa were reported, belonging to 63 families (Sáfián et al. 2009; De Prins and De Prins 2019). The currently known number of species in Kenya is almost twice that of Ethiopia, and 15 families are not recorded at all in the latter country. Is it really due to difference in faunal richness between the two coutries or because of the different level of investigation? A better idea can come from the differences observed within the single families. When considering most groups of the ‘Microlepidoptera’, very few investigations were made in Ethiopia and the difference in species numbers between the two countries is huge. Considering only the most species-rich families of Microlepidoptera, the percentage of species present in Ethiopia, compared to the species numbers in Kenya, is 10% for Scytrididae, 13% for Gelechiidae, 17% for Thyrididae, 31% for Tortricidae, 34% for Pyralidae, 45% for Crambidae, 46% of Pterophoridae, up to 76% for Tineidae. However, if we look at the ‘larger moths’ (Macroheterocera) and butterflies, which are better investigated in both countries, the difference is decreasing from 40% for Saturniidae, 41% for Geometridae, 50% for Lycaenidae, 53% for Sphingidae, 55% for Erebidae up to 77% for Papilionidae, 79% for Noctuidae, 91% for Nymphalidae, peaking to 132% in Pieridae, where Ethiopia shows a higher number of species than Kenya.

Although the two countries certainly exhibit faunistic differences, due to biogeographic or climatic factors, it seems clear that the Ethiopian fauna is seriously understudied in many groups. By analysing comprehensive revisions of single genera or families accompanied by major collection campaigns in Ethiopia, we can have an idea of the potential biodiversity the country inhabits.

The geometrid genus *Prasinocima* Warren, 1897 was subject of an extensive review focused on Ethiopian species, based on an investigation carried out in 100 collection localities in the country for more than 15 years, which included an integrative taxonomic analysis based on morphology and DNA barcodes (Hausmann et al. 2016). As a result of this contribution, the species number was raised from eight previously known Ethiopian species to 40, of which 19 were new to science. After the publication, another seven new species for the Ethiopian fauna were described. Authors of the same article estimated the number of Ethiopian geometrids to exceed 700 species once the unidentified material in their hands is examined, which may suggest a more realistic total species number in excess of 1,000 for the whole country.

Another contribution came from the revision that Hacker carried out on the subfamily Nolinae (Nolidae; Hacker et al. 2012; Hacker 2014), where many of the published data concerned sub-Saharan Africa. For Ethiopia, only three species were previously reported. After Hacker’s monograph, the number was raised to 61 species, with 27 newly described taxa from Ethiopia. For Kenya, he raised the figure from 12 to 73, a number not far from that of Ethiopia.

Although these are two examples of taxonomically particularly difficult groups, we can assume similar multiplicators for the so called ‘Microlepidoptera’ resulting in an estimate for the entire order of Lepidoptera in Ethiopia which may exceed 10,000 species, of which a number of species new for science. This estimate is based on, and in concordance with the usual ratio of geometrid species number versus lepidopteran species number of roughly 1:10, and on the usual ratio of the Rhopalocera (400+ species in Ethiopia) versus lepidopteran species number of roughly 1:20, as it results from large museum material (e.g. ZSM) and from various fauna inventories (e.g. Bavaria: Haslberger and Segerer 2016; Europe: Karsholt and Razowski 1996; North America: Hodges et al. 1983). For the moth fauna of Africa, 38,988 species group names of them are listed by Afromoths (2019), of which 5510 (14%) are geometrids. The total number, however, does not include Rhopalocera names, with 4405 species (Williams, 2018) and Microlepidoptera taxonomy is underrepresented, hence also here the “10%-rule” for the Geometridae ratio seems to apply, at least roughly.

## Data from DNA barcoding

In the framework of the international Barcode of Life initiative, DNA barcodes (658bp 5’ COI gene fragment, cf. Hebert et al. 2003) have been assembled for Ethiopian Lepidoptera since 2006 with the aim to establish a national DNA reference library for integrated taxonomic studies. So far, 3160 DNA barcodes have been generated from Ethiopian Lepidoptera (including many Ethiopian type specimens), belonging to 1012 genetic clusters (Barcode Index Numbers, ‘BINs’) which are a good proxy for real species numbers (Ratnasingham and Hebert 2013; Hausmann et al. 2013). Most DNA barcodes could be assembled in the Geometridae (2290 barcodes, 571 BINs), Noctuidae (314 barcodes, 165 BINs) and Erebidae (246 barcodes, 143 BINs). Species coverage is particulary good in the smaller families such as the Saturniidae (121 barcodes, 36 BINs) and Sphingidae (70 barcodes, 24 BINs), while it is still being very poor in the ‘Microlepidoptera’. All images and most metadata and molecular data are accessible in the public database BOLD (Ratnasingham and Hebert 2007).

## Actual constraints and future perspectives of research on Lepidoptera Diversity of Ethiopia

Butterﬂies and moths are a major component of biodiversity playing a crucial role in the ecosystem as primary consumers, essential part of food-chains and pollinators. However, humans are exerting unprecedented pressures on all of the earth’s ecosystems, and such pressures may aﬀect all species (Sanchez-Bayo and Wyckhuyes 2019). Nature conservation strategies have focused most of their attention on the “charismatic megafauna”, i.e., on mammals, birds, and other vertebrates. The vast majority of invertebrate species – although accounting for more than 80% of the animal species - are too poorly known to allow an assessment of how they are aﬀected by human activities, and what might be done to mitigate the damage that humans cause. In most cases, the best way that can be done is to conserve their habitats so that most inhabiting species will continue to thrive.

The greatest threats to butterﬂies and moths are habitat fragmentation and destruction, intensification of agricultural practice with over-use of pesticides and herbicides; climate change mainly affecting endemic species adapted to mountainous habitats, whereas scientific collecting is absolutely negligible (Hausmann 2001; Sanchez-Bayo and Wyckhuyes 2019). In general, human activity is enormously threatening the global diversity of life on the planet. Rough estimates suggest that we are currently undergoing not only unprecedented, but also accelerating rates of species extinction (UNEP 2006; Sanchez-Bayo and Wyckhuyes 2019).

In the same manner, Ethiopia is experiencing major biodiversity loss, mainly related to extensive destruction of habitats, deforestation, land degradation, intensive agricultural expansion, climate change, excessive pesticide and herbicide use, introduction of exotic plant species, among others (EBI 2015; Tesfu et al. 2018). The loss of primary or native forest areas, due to clearcutting and conversion into agroforests, farmland or settlements, are currently the major threat to the Ethiopian biodiversity in general and Lepidoptera in particular.

Despite Ethiopia being known for its rich heritage of biological diversity and many diverse ecosystems, the conservation of its habitats have received scant attention. The system of protected areas so far established includes 21 national parks, four sanctuaries, eight wildlife reserves, 20 controlled hunting areas, six open hunting areas, six community conservation areas and 58 national forest priority areas (Young 2012), covering 14% of the country (EBI 2015). However, most of its biodiversity, including Lepidoptera, is still unexplored because of significant lack of national research capacity. Hence, in parallel to conservation programs and sustainable utilisation of biological resources, efforts for the preparation of a comprehensive bio-inventory should receive highest priority. Such an instrument must be considered an essential baseline for policy makers, planners, donors and researchers working on biodiversity conservation in Ethiopia.

In order to upsurge biodiversity knowledge, capacity building in the area at various levels is needed. Lack of well organised natural history museums, specialists, and scientific societies providing support and fostering citizen science, international research networks and projects are among the identified gaps. Currently, most of the type specimens and reference collections are deposited outside the country of origin. In this context, the Nagoya Protocol (UNSG 2010), although intending to strengthen nations to conserve their genetic resources, to some extent could lead to the opposite effect by hampering international collaboration. Joint protocols and agreements between national actors (research institutes, governing agencies, universities, NGO’s) and international research bodies should be promoted in a collaborative way, favoring shared, non-commercial biodiversity research. Close collaboration with museums and universities possessing reference collections and skills, designing and organising projects are required to teach and train a generation of highly competent scientists and managers so that collections of Ethiopian insects could be built and properly managed. In absence of these minimum requirements, establishing a national entomological museum/collection could be ineffective in promoting the study and conservation of local biodiversity resources.
